# An Evaluation of Aluminum Tolerant *Pseudomonas aeruginosa* A7 for *In Vivo* Suppression of Fusarium Wilt of Chickpea Caused by *Fusarium oxysporum* f. sp. *ciceris* and Growth Promotion of Chickpea

**DOI:** 10.3390/microorganisms10030568

**Published:** 2022-03-05

**Authors:** Atifa Begum Mozumder, Kakoli Chanda, Ringhoilal Chorei, Himanshu Kishore Prasad

**Affiliations:** Department of Life Science and Bioinformatics, Assam University, Silchar 788011, India; atifamozumder@gmail.com (A.B.M.); kakolimic@gmail.com (K.C.); choreiringhoilal@gmail.com (R.C.)

**Keywords:** *Fusarium oxysporum* f. sp. *ciceris*, aluminum, biocontrol, chickpea, PGPR, *Pseudomonas aeruginosa* A7

## Abstract

Chickpea wilt, caused by *Fusarium oxysporum* f. sp. *ciceris*, is a disease that decreases chickpea productivity and quality and can reduce its yield by as much as 15%. A newly isolated, moss rhizoid-associated *Pseudomonas aeruginosa* strain A7, demonstrated strong inhibition of *Fusarium oxysporum* f. sp. *ciceris* growth. An *in vitro* antimicrobial assay revealed A7 to suppress the growth of several fungal and bacterial plant pathogens by secreting secondary metabolites and by producing volatile compounds. In an *in vivo* pot experiment with Fusarium wilt infection in chickpea, the antagonist A7 exhibited a disease reduction by 77 ± 1.5%, and significantly reduced the disease incidence and severity indexes. Furthermore, A7 promoted chickpea growth in terms of root and shoot length and dry biomass during pot assay. The strain exhibited several traits associated with plant growth promotion, extracellular enzymatic production, and stress tolerance. Under aluminum stress conditions, in vitro growth of chickpea plants by A7 resulted in a significant increase in root length and plant biomass production. Additionally, hallmark genes for antibiotics production were identified in A7. The methanol extract of strain A7 demonstrated antimicrobial activity, leading to the identification of various antimicrobial compounds based on retention time and molecular weight. These findings strongly suggest that the strain’s significant biocontrol potential and plant growth enhancement could be a potential environmentally friendly process in agricultural crop production.

## 1. Introduction

Chickpeas (*Cicer arietinum* L.) are considered to be one of the oldest legumes known from ancient times. Most likely, it originated in southeastern Turkey and Syria’s adjacent areas [1]. Globally, it is a valuable crop and about 2.3 million tons enter the world market annually [2]. The major chickpea legume grain-producing regions are in southern and South-Eastern Asia, Africa, and Australia, [2].

It is estimated that India produces 9.075 million tons of chickpeas annually, accounting for 65% of the total production of chickpeas. The remarkable growth of chickpea pulse has been documented in India, comprising the most significant production (47%; 10.90 MT) among all pulse crops in 2019–2020 [2]. Chickpeas are sustainable legumes with nutritional value for human and animal consumption as well as soil fertility. Chickpeas seeds and leaves are an excellent source of carbohydrates, vitamins, fibers, and proteins. Chickpeas play a leading role in food safety in the world by supplying the protein deficit of daily food rations [3]. Chickpeas-based diets are recommended to combat lifestyle disease since they contain several essential minerals such as calcium, phosphorus, iron, and zinc among other substances, including phenolics and oligosaccharides [3]. There are numerous living and non-living stressors that affect chickpea production globally [4,5]. Among them, Fusarium wilt is the most severe biotic stressor of chickpeas, caused by *Fusarium oxysporum* f. sp. *ciceris* (*Foc*) in chickpea-growing nations all around the world [5,6,7]. *Foc* is a significant limiting factor in chickpea production, causing yield losses ranging from 10 to 100% depending on variety and climatic conditions [8,9]. This plant disease exhibits symptoms such as wilting, drooping, discoloration, yellowing, browning of the xylem vessels, and leads to the eventual collapse of the whole plant [6,9]. In chickpea, several sources of resistance have been identified [10,11], which have been successfully used in resistance-breeding programs at the Indian National Agricultural Research System (NARS) and International Crops Research Institute for the Semi-Arid Tropics (ICRISAT), increasing chickpea production [12]. A total of 8 *Foc* pathogenic race variants have been identified in the world so far (races 0, 1A, 1B/C, 2, 3, 4, and 6) [11,13]. Biological control of *Foc* using rhizobacteria is an ecologically sustainable approach since the pathogen is soil-borne and the indiscriminate chemical control measures lead to increased pesticide tolerance [13,14,15,16]. Several pseudomonas species found in the rhizosphere have proven to be effective biocontrol agents, both due to their widespread distribution in soil and their production of heat-resistant spores [17]. In agriculture, plant pathogenic fungi are responsible for severe damage and the loss of agricultural products [18,19]. The antagonist Plant Growth-Promoting Rhizobacteria (PGPR) act as biocontrol agents (BCAs) and secrete active ingredients such as natural products [20]. Natural products secreted by bacteria may suppress pathogen growth either directly by producing volatile or non-volatile secondary metabolites or indirectly by participating in the competition for nutrients [21,22,23]. Plant growth-promoting biocontrol agents produce natural products such as pyrrolnitrin, phenazine, phloroglucinol, hydrogen cyanides, siderophores, hydrolytic enzymes that inhibit and suppress the plant pathogen *Fusarium oxysporum* [23,24,25]. Additionally, *Trichoderma harzianum* and *Bacillus subtillis* secrete β-1,3 glucanase enzymes that have an inhibitory effect on chickpea wilt by inhibiting the pathogen’s growth [23,26]. Volatile organic compounds (VOCs) produced by PGPBs suppress phytopathogenic growth and produce morphological and physiological changes to plants, such as an increase in chlorophyll content, which further improves plant growth [27].

Plant growth-promoting bacteria (PGPB) that can withstand metals are being used to develop the growth of numerous crops in metal-polluted environments [28]. Microbes can be used in the agricultural field to protect natural habitats and ensure healthy crop production. *Burkholderia ginsengiterrae* N11-2, *Chryseobacterium polytrichastri* N10, *Pseudomonas fluorescens, Pseudomonas fragi* N8 and *Pseudomonas simiae* N3, have all been shown to grow in aluminum-stress conditions [28,29]. Plant Growth-Promoting Rhizobacteria (PGPR), which can produce phytohormones that are essential for plant growth, interact with the flora by increasing nutrient accessibility [21,23,30]. The pseudomonas strains can promote plant growth, as well as advance plant responses to harsh conditions, and allocate all the important characteristics to the host plant to promote growth [31,32,33,34,35]. North-Eastern India has various agro-climatic zones that demand a range of cultivation methods. The excessive rainfall in this region is causing basic cations in the soil in the region to leach out over time, destroying soil texture and bringing the thriving of metals like aluminum to bear on crop health [36]. Chemical forms of aluminum exhibit the highest degree of toxicity to plants when exposed to pH < 5 [37]. The metal has gained attention due to its toxic effects on acidifying soil, which limits crop production worldwide and the growth of most living organisms [38,39]. Aluminum’s toxicity has become a major concern as this metal is usually formed into complexes with silicates liberated because of acid rain [40]. Aluminum (Al) levels, about 1ppm in soil, reduce root growth through limiting water and nutrient uptake, which affects plant health and crop productivity [36,41]. By increasing the expression of aluminum stress-related genes, PGPBs are shown to improve the plant’s tolerance mechanisms [28,42]. *Pseudomonas fluorescens* (ICA/BECa 141), *Kosakoniar adicincitans* (IAC/BECa 95), *Paraburkholderia tropica* (IAC/BECa 135), and *Herbaspirillium frisigense* (IAC/BECa 152) have been shown to increase sugarcane growth on high Al bioavailability and nutrient as well as Al uptake in crop plants under acid soil conditions [43]. *Pseudomonas plecoglossicida* Pp20 strain ameliorates damages on maize roots induced by aluminum chloride (AlCl_3_) at a concentration of 90 µM by producing the plant growth-promoting hormone indole-3-acetic acid (IAA) and ethylene degrading enzyme, 1-aminocyclopropane-1-carboxylic acid (ACC) deaminase [44]. Apart from the production of phytohormones, the PGPB may evolve diverse mechanisms such as mineral solubilization, the development of siderophores (metal-chelating compounds), inhibiting metal transport in the roots, reducing lipid peroxidation and hydrogen-peroxide production, and sustained enhancement of antioxidant enzymes in the plants [41,42,43,44,45]. Few reports are available for chickpea metal tolerance using PGPB under salinity and metal (Mn) toxicity conditions [46,47]. Combining endophytic bacteria (*Stenotrophomonas maltophilia*), mycorrhizal fungi, and ferrimagnetic magnetite (Fe_3_O_4_) nanoparticles induced *Rhizobium* improved chickpea (*Cicer arietinum* L.) nodulation, leg hemoglobin content, nitrogenase activity, and growth by mitigating salt and metal stress and improving nutritional status of plants at salinity levels 75 and 150 mM [47]. However, no attempt has been made to employ PGPR inoculation to mitigate Al stress in chickpea plants growing in acid soil with high Al levels. The current study reports on the identification and characterization of a *Pseudomonas aeruginosa* strain A7 isolated from rhizoids of *Polytrichum* sp. from a waste disposal area. We report its biocontrol potential against phytopathogens and characterize its abilities to produce plant-growth promotion-related compounds such as indole-3-acetic (IAA), ammonia, siderophores, hydrogen cyanide (HCN), cellulase, chitinase, pectinase, proteases, the ability to solubilize tri-calcium phosphate, potassium, zinc oxide and can tolerate different abiotic stress conditions. The strain A7 enhanced the chickpea growth in aluminum stressed conditions and acts as a potential biocontrol agent to Fusarium wilt disease of chickpea caused by *Fusarium oxysporum* f. sp. *ciceris* (*Foc*).

## 2. Materials and Methods

### 2.1. The Isolation, Morphological and Biochemical Characterization of Aluminum-Tolerant Bacteria from the Rhizoids of Polytrichum sp.

Adult moss plants identified as *Polytrichum* sp. were collected from a waste disposal area at Cachar Paper Mill Panchgram, Hailakandi, (Assam), India. Approximately 10 g of tiny rhizoids of the moss adhering with soil were cut out and mixed with 90 mL phosphate-buffered saline (PBS) (HiMedia, Mumbai, India) in a conical flask. The flask was kept in a rotary shaker incubator at 150 rpm at 30 ± 2 °C for 60 min. Following shaking, the suspension was serially diluted to a factor of 10^−6^ with Phosphate-buffer-saline (PBS) buffer and plated on King’s Base agar medium (KB) (HiMedia, Mumbai, India) plates supplemented with aluminum (AlCl_3_, SD Fine Chemicals, Mumbai, India) and incubated for 2 days at 30 ± 2 °C [48,49]. On the plates, bacteriologically distinct colonies were selected for further purification. Pure strains were preserved at −80 °C in 20% glycerol [50]. A strain designated A7 was studied for its morphological characteristics, such as color, shape, size, elevation, and surface. The fluorescent activity of the colonies that appeared under ultra-violet (UV) light on the plates was evaluated after 24 h by re-inoculating them on the KB plate. The strain was processed through different biochemical characterizations such as catalase, citrate utilization, indole synthesis, methyl red generation, nitrate reduction, starch hydrolysis, urease, Voges-Proskauer, and hydrogen sulfide (H_2_S) production was achieved using KB003 Hi25TM *Enterobacteriaceae* identification Kit (HiMedia, Mumbai, India) [51].

### 2.2. Molecular Identification of the Strain

The 16S rRNA-encoding gene sequencing-based molecular identification of the strain was performed using total genomic DNA [52]. For the amplification of the 16S rRNA gene fragment, the fD1 (5′-GAGTTTGATCCTGGCTCA-3′) and rP2 (5′-ACGGCTACCTTGTTACGACTT-3′) primer set was used [53]. Polymerase chain reaction (PCR) mixture (50 µL) contained 50 ng of genomic DNA template, 0.5 µM of each of the primers, 200 µM of dNTPs, 1X-High-Fidelity buffer, 1U of Phusion High-Fidelity DNA polymerase enzyme (Thermo Scientific, Waltham, MA, USA). The polymerase chain reaction amplification was performed using a C1000 Touch Thermal Cycler (Bio-Rad, Hercules, CA, USA) with cycle conditions of initial denaturation at 98 °C for 30 s, 35 cycles of initial denaturation at 98 °C for 10 s, 50 °C (annealing) for 30 s, 72 °C (extension) for 1 min 30 s, and a final extension of 72 °C for 7 min. Gel electrophoresis was performed on the amplified products using 1.5% agarose and Tris-Acetate-EDTA (TAE). The PCR product was purified using a QIAquick PCR purification kit (Qiagen, Hilden, Germany). The restriction enzyme *EcoRV* (NEB, Ipswitch, MA, USA) cut plasmid vector pLitmus28 (NEB, Ipswitch, MA, USA) and 16S rRNA DNA was blunt ligated with T4 DNA ligase (Thermo Scientific, Waltham, MA, USA) to clone the PCR product. Electrocompetent *E. coli* TOP10 cells (Thermo Scientific, Waltham, MA, USA) were prepared and transformed with a ligation mix using a Gene Pulser (Bio-Rad, Hercules, CA, USA), following the standard protocol [54]. Using the corresponding primer set, colonies on Luria Bertani (LB) agar fortified with Ampicillin 100 µg mL^−1^ were selected and confirmed by colony PCR and plasmid DNA was isolated. An *EcoRI* restriction digestion (Thermo Scientific, Waltham, MA, USA) was performed to obtain the product of the desired size of 4.3 kb. The recombinant DNA products were purified further using PEG 8000 (Sigma Aldrich, Bangalore, India) and sequenced commercially. Two reads of the amplicon sequencing were assembled and analyzed using Chromas Pro version 2 (http://technelysium.com.au/wp/chromaspro/, accessed on 25 July 2020). To identify the strain, a blastn analysis was performed by comparing the derived gene sequence to known bacterial 16S ribosomal RNA gene sequences obtained from the NCBI GenBank website (https://www.ncbi.nlm.nih.gov/, accessed on 25 July 2020). A phylogenetic analysis was performed with the Maximum Likelihood method with 1000 bootstrap replications using MEGA version 6.0 software (https://www.megasoftware.net/, accessed on 25 July 2020) [55]. This nucleotide sequence was submitted for inclusion in GenBank under accession number MN197639.

### 2.3. In Vitro Antagonistic Activity of the Strain A7

To assess the in vitro antimicrobial activity, plant pathogenic fungal strains *Rosellinia arcuata* Petch (*Rap*) (ITCC 4143), *Poria hypobrunnea* Patch (*Php*) (ITCC 4141), *Ustillinia zonata* (Lev.) Sacco (*Uzs*) (ITCC 4144), *Phellinus lamaensis* (Murrill) Pat. (*Plp*) (ITCC 4140), *Fusarium oxysporum* (*Fo*) (ITCC 7093), *Fusarium oxysporum* f. sp. *ciceris* (*Foc*) (ITCC 3636) *Fusarium oxysporum* f. sp. *lycopersici* (Sacc.) (*Fol*) (ITCC 1322), *Fusarium oxysporum* f. sp. *pisi* (*Fop*) (ITCC 4814), *Fusarium solani* (*Fs*) (ITCC 6953), *Curvularia* *eragostidies* (*Ce*) (ITCC 5540) and *Curvularia lunata* (Wakker) Boedijn var. (*Clb*) (ITCC 5193) were obtained from Indian Type Culture Collection (ITCC), in the Division of Plant Pathology, Indian Agricultural Research Institute, New Delhi. The fungal pathogen *Rhizoctonia solani* (*Rs*) and bacterial pathogens *Ralstonia solanacearum* (*Ras*), *Xanthomonas campestris* (*Xc*) were kindly supplied from Assam Agricultural University, Jorhat, India.

The in vitro antifungal activity of strain A7 was performed using a concentric ring bioassay [56]. In the center of the Potato-dextrose Agar (PDA) plate, a 6 mm diameter mycelium disc was cut from the margin of a 4-day-old PDA grown fungal mycelium and placed. The bacterial strain was inoculated surrounding the fungal disc in a concentric ring manner with the help of a sterile funnel. As a control, a fungal disc without a concentric ring of bacteria was employed. The percentage of inhibition was calculated after 2 to 7 days of growth suppression at 30 °C, depending on the pace of growth. Using the formula below, the percentage of growth inhibition (PGI) of the fungal pathogen by the strain was calculated.

The dual-culture assay was used to produce antifungal volatile organic compounds (VOCs) [57]. A King’s B agar plate was streaked with strain A7, and a fungal mycelium disc was placed onto the center of the PDA plate. Parafilm was used to seal both inoculation plates [58]. However, it should be emphasized that the Petri dishes create a gap in the growth media, preventing the nonvolatile exudates secreted by A7 from reaching the fungus. KB plates spread with sterile distilled water and the fungal disc on a PDA plate sealed with parafilm was considered a control. The growth inhibition was determined after 2 to 7 days at 30 ± 2 °C, depending on their growth rate and the percentage of growth inhibition was recorded. The percentage of growth inhibition (PGI) of the fungal pathogen by the strain was calculated as:

(1) $$\mathrm{PGI}\;\left(\%\right)=\left(D_{c}-{\;D}_{t}\right)/\left(D_{c}\right)\times 100$$

where D_c_ is the pathogen’s growth rate in the absence of strain A7, and D_t_ is the pathogen’s growth rate in the presence of strain A7.

*In vitro* antifungal and antibacterial activities by the strain A7 with the cell-free supernatant (CFS) were completed by Agar-well diffusion and the Agar disk-diffusion method [59]. To establish the stationary phase cultures, A7 was cultured on KB broth for 2 days at 30 °C. The cell-free supernatant of the strain A7 was collected by centrifugation at 13,000 rpm for 10 min at 28 °C. To eliminate any debris, CFS was filtered using a 0.2 µm membrane filter (Millipore) and was ready to use [60]. Antifungal activity by using CFS was performed by inoculating a fungal disc at one edge of the PDA plate. A rectangular well was prepared at another edge using a sterile scalpel and a volume of 100 µL of CFS was introduced into it. The Petri dishes were incubated for 48 h at room temperature. The CFS was diffused into the medium and the growth of the fungal pathogen was inhibited and measured as above.

Antibacterial activity using the CFS of the strain A7 on KB agar plates was performed by inoculating freshly grown bacterial pathogens (0.1 optical density at 600 nm, OD_600_) by streaking. Then, on both ends of the agar surface, filter paper discs (6 mm in diameter) were inserted and labeled accordingly (i) filter paper disc with 50 µL CFS, (ii) filter paper disc with 50 µL sterile double distilled water as control. The Petri dishes were incubated at 30 ± 2 °C for a day. CFS was diffused into the medium and the germination and growth suppression of the respective pathogen was calculated by measuring the diameters of the zone of inhibition [61]. All assays were performed in three biological replicates.

### 2.4. Plant Growth-Promoting (PGP) Activities of the Strain A7

The strain’s plant-growth-promoting activities were assessed qualitatively using a plate-based assay and quantitatively using a liquid assay. Luria-Bertani (LB) agar plates amended with 5 mM L-tryptophan (SRL, Mumbai, India), Sodium Dodecyl Sulfate (SDS, 0.06%), and glycerol (1%), was used to detect Indole-3-Acetic acid (IAA) production. To obtain a colony with a diameter of 0.2–5 mm, the plates were incubated for 24 h. After that, the Petri dishes were covered using Whatman no.1, filter paper, and incubated for another 24 h. The filter papers were removed after the incubation period, saturated with Salkowski reagent (4 mL prepared using 2% of 0.5 M Ferric III Chloride in 35% of Sodium hypochlorite), and studied at 30 °C for 1 to 2 h until appropriate color development. The development of the red halo zone around the colony on the filter paper was considered a positive IAA producer [62]. IAA production was also performed quantitatively by inoculating the strain on the LB broth using Salkowski reagent with a 48 h time interval of 196 h. The absorbance was measured at 530 nm after the development of the pink color. Using the IAA standard (LobaChemi, Mumbai, India), the concentration of the generated IAA was measured [62]. Phosphate was solubilized on Pikovskaya’s (Pkv) agar (HiMedia, Mumbai, India), using the method outlined in [63]. Quantitative estimation was performed on the Pkv broth medium by the method described [64]. Briefly, 25 mL Pkv broth was inoculated with A7 and incubated at room temperature for 4 days. Additionally, 1 mL of the bacterial cell-free supernatant prepared by centrifuging at 30 min at 12,000 rpm, (Eppendorf Centrifuge 5804R, Eppendorf, Hamburg, Germany) was mixed with 10 mL chloromolybdic acid, and 35 mL of distilled water was added. The volume was then increased by adding chloro-stannous acid (0.25 mL). The total volume of 50 mL was maintained by adding distilled water. The absorbance was measured at 600 nm of the developing blue solution. The quantity of soluble phosphorus was determined using the KH_2_PO_4_ standard curve (Sigma Aldrich, St. Louis, MO, USA). A modified plate assay was used to determine the solubilization of the potassium [65] by inoculating the strain A7 on Aleksandrov agar medium mixed with Bromothymol Blue (an acid-base indicator dye) [66]. The Petri dishes were incubated at room temperature for 7 days. An improved visibility solubilization halo zone on the agar plates was positive for potassium solubilization. The formula for calculating the solubilization efficacy (SE) was as follows: SE = [the diameter of A7/the diameter of the halo zone] ∗ 100 [67]. By inoculating the strain on zinc oxide agar medium plates and incubating them at 30 °C for seven days, zinc oxide solubilization was measured qualitatively [68]. The above formula was used to compute the solubilization efficiency. The strain A7′s ability to fix atmospheric N_2_ was investigated using the nitrogen-free broth (Nfb) medium described [69]. At 30 °C, the plates were incubated for 24 h. After the incubation period, the medium’s color changed from pale green to blue, indicating the isolate’s ability to fix N_2_. Ammonia was produced by growing A7 on peptone water (10 mL) in each falcon tube and incubated for 2 to 3 days at 30 ± 2 °C. Following incubation, 500 µL of Nessler’s reagent was added to each tube. Brown to yellow color development was taken as a positive test for the production of ammonia [70]. Chrome Azurol S (CAS) assays were used for siderophores production [71]. The freshly prepared CAS solution was added to the KB agar medium as a 1:15 ratio and sterilized by autoclaving. Plates inoculated with A7, showing an orange halo zone surrounding the colony at 30 ± 2 °C for 3 days of incubation, were considered positive for siderophores production, and the diameter of the colored zone was recorded. The A7 was inoculated on a 20 mL KB medium and incubated at the above-mentioned parameters to determine the production of siderophores. Centrifugation at 3000 rpm for 5 min was used to remove the cells. Furthermore, 3 mL of cell-free supernatant, 300 µL of 5N hydrochloric acid solution, 1.5 mL of Arnow’s reagent, and 300 µL of 10N NaOH were mixed. The formation of a pink color was noticed after 10 min and recorded. The KB medium amended with 4.4 g of glycine/L was used to test the production of HCN using the strain A7. The socked filter paper (Whatman no. 1, Ankleshwar, India) on 2% Na_2_CO_3_ in the picric acid solution (0.5%) was kept on top of the Petri dishes. The dishes were packed with black paper after sealing with parafilm and incubated at 30 ± 2 °C for 4 days. HCN production was detected by the hue changing from yellow to red brown [72]. The chitinolytic activity was assayed on the chitin agar plate, as described by [73] with some modification. In Petri dishes, the lukewarm medium was poured and was allowed to harden. The A7 was inoculated onto the medium and incubated at 30 °C, where it was monitored for the formation of a purple halo zone surrounding the colony, and the measurement of the purple halo was recorded. The polyamine production using A7 was tested by streaking on a Long Ashton Decarboxylase (LAD) agar [74] for 7 days, the Petri dishes were incubated in the dark at room temperature. The strain’s arginine decarboxylation was represented by red halos on a yellow backdrop. The extracellular enzymatic activity of strain A7 was using different modified plate-based methods. Cellulase and xylanase activity was estimated by inoculating the strain on Congo-red gelatin agar medium supplemented with 0.5% Carboxymethylcellulose (Na-CMC, Sigma Aldrich, St. Louis, MO, USA) and xylan (Beechwood Xylan, Sigma Aldrich, St. Louis, MO, USA), respectively [75]. The Petri dishes were incubated for 3 to 7 days at room temperature. Bacterial colonies stained with congo-red were thought to be cellulose and xylan degraders [76]. The positive strain’s degrading potential was also measured qualitatively by equating hydrolysis capacity (HC). HC = Ratio of the halo zone diameter to the colony diameter [77]. The ligninolytic activity was observed using a Minimal Salt Medium-Kraft Lignin (MSM-KL) medium containing 0.5% Alkali lignin (Sigma Aldrich, St. Louis, MO, USA) supplemented with Methylene blue as the lignin polymeric dye and incubated at 30 ± 2 °C for 3 to 7 days. The strain possesses ligninolytic enzymes that undergo the oxidation of indicator dye. The plates were checked daily for bacterial growth and Methylene blue dye decolorization [78]. Pectinase production was estimated by inoculating A7 on an MM Pectin agar medium supplemented with 0.5% Citrus Peel Pectin (Sigma Aldrich, St. Louis, MO, USA) [79]. The Petri dishes were incubated for 2 days at room temperature and flooded for 15 min with a 5.0% (*w*/*v*) iodine solution (LabChem, India) to detect the halo zone around the colony, which shows the strain’s ability to produce pectinase. Proteases activity was performed by inoculating A7 on a Skim milk agar medium supplemented with 0.5% skim milk (Sigma Aldrich, St. Louis, MO, USA) and tested positive by the development of a distinct halo zone around the colony [80]. The above-mentioned formula was used to calculate the semi-quantitative enzyme production.

### 2.5. Growth Profile of Strain A7 on Different Concentrations of Aluminum, Iron, and Other Stressed Conditions

The growth profile of strain A7 was checked to tolerate an increasing concentration of aluminum (AlCl_3_) (0–4 mM) and other metal compounds using the Single Plate Serial Dilution Spotting (SP-SDS) method on modified M9 agar plates [80]. The liquid culture was in the M9 broth supplemented with 0.1% proteases peptone. Similarly, the effect of Fe (III) from 0–5 mM, Fe (II) from 0–3 mM, NaCl (0–8%) (Sigma Aldrich, St. Louis, MO, USA) [81], pH (1–14) and temperature (4–50 °C) was checked in three biological replicates [82,83]. Freshly grown bacterial cells (0.1 OD_600_) were serially diluted from 10^−1^ to 10^−6^, and 3 µL from each dilution was inoculated into the demarcated areas of SP-SDP and in a 10^−1^ dilution in a 50 mL broth. The Petri dishes were incubated for 18–48 h, and the flask was held in an orbital shaker at 160 rpm at 30 ± 2 °C for 48 h. The optical density of the cultures was taken at 600 nm (PerkinElmer UV/VIS spectrometer Lamda 25, USA). The relative growth (RG) was calculated using the formula [84]. Relative Growth (%) = A_t_/A_c_ ∗ 100, where A_c_ = optical density of the average of three biological replicates of controls, and A_t_ = optical density of average three biological replicates of the treatments. Bacterial 20% and 50% growth inhibitory concentration (IC) IC_20_ and IC_50_ values were calculated using the dose–response data.

### 2.6. In Vitro Protective Effect of Strain A7 on Aluminum Toxicity in Chickpea Growth

For the *in vitro* protective effect of strain A7 against aluminum toxicity in chickpea, growth was verified using the jar method [36]. An adequate quantity of chickpea seeds (Pusa-362) was surface-sterilized for 3–5 min in a 0.1% HgCl_2_ solution followed by 6–7 washing with sterilized distilled water. The seeds were saturated in water for 12 h after which they were kept on moisten filter paper placed within a Petri dish for germination at 28 ± 2 °C for 72 h. The healthy germinated seeds of equal root and shoot length were transferred into a plastic jar filled with 400 mL of Hoagland nutritive solution (HiMedia, Mumbai, India). Over a 14 h photoperiod, plantlets were cultivated in a plant-growing room in white light with a photon flux intensity of 220 mol m^−2^ s^1^ (PAR). The Hoagland nutritive solution changed every 48 h to maintain the seedlings proper growth. These plantlets were then pretreated with 500 µM CaCl_2_ (pH 5) for a day. The growth of strain A7 was previously checked on different concentrations of CaCl_2_ (100, 200, 500, and 1000 µM). After one day, the pretreated seedlings were transferred to an experimental setup (1) T_0_ = Control (with no bacteria and no aluminum), (2) T_1_ = AlCl_3_ (only 100 µM of aluminum chloride), (3) T_2_ = A7 (only 0.1 OD_600_ cells of the bacterial strain) and (4) T_3_ = A7 + AlCl_3_ (0.1 OD_600_ cells of the bacterial strain + 100 µM of aluminum chloride) for 12, 24, 36, 48 h. The root length and shoot length, root fresh weight and dry weight, and relative root length rate (RRL) were all assessed to determine total growth and biomass. Four plants from each treatment were randomly selected and root shoot length was measured. For plant biomass measurements, four plants were grouped to measure the fresh weight and were dried at 80 °C for 48 h, weighing the dry biomass. Seed vigor index (SVI) [85] and relative root length (RRL) rate [36] were calculated using the formulae:

(2) SVI =(Shoot length + root length)× seed germination%

(3) RRL =(Root length in aluminum stress conditions) / (Root length in control conditions)×100

### 2.7. Control of Fusarium Wilt of Chickpea by Strain A7 in Pot Assay Experiment

The chickpea seeds of variety (Pusa-362) were collected from Krishi Vigyan Kendra, Hailakandi, Assam, India, and were used in the pot experiments. The variety Pusa-362 was tested for Fusarium wilt-disease control in pot and field experiments using bacterial biocontrol agents [86]. *Fusarium oxysporum* f. sp. *ciceris* (*Foc*) conidia were prepared by inoculating the pathogen onto the PDA medium incubated at room temperature for 8 days. For a healthier spore suspension, actively growing margins on the culture media plate was removed and 4 to 5 mL sterile dH_2_O was added. The mycelia were separated from the spore suspension, the conidial suspension was harvested, and the final concentration of the inoculum was prepared at 10^6^ conidia/mL. Bacterial inoculum was prepared by growing the strain A7 in KB broth, 30 °C, 160 rpm, for a day. The cell culture was centrifuged for 10 min at 6000 rpm and a fresh pellet, the suspension was prepared in one-fourth strength of 1% sterile carboxymethylcellulose (Sigma Aldrich, St. Louis, MO, USA) to prepare a final concentration of 10^8^ bacteria/mL [87].

Dry vermiculite soil collected from the vegetable field was crushed, sieved, and sterilized by autoclave. The seeds were surface-sterilized for 4–5 min with an HgCl_2_ (0.1%) solution, then washed 6–7 times with sterile dH_2_O. The seeds were saturated in sterile water for 12 h, transferred, and placed properly in a seedling bed prepared with the soil in a plastic tray for germinated and grown at 28 ± 2 °C for 7 days. Seven numbers of 7 days old seedlings were then transferred to a pot containing 2 kg of soil.

The seedlings were treated by inoculating 5 mL of bacterial cell suspension and 5 mL fungal spore suspension in the following treatments: (1) T_0_ = sterile distilled water as control (2) T_1_ = only bacterial cell suspension (3) T_2_ = only fungal spore suspension, (4) T_3_ = bacterial cell suspension and fungal spore suspension, (5) T_4_ = bacterial cell suspension two days before the fungal spore suspension, (6) T_5_ = fungal spore suspension two days before the bacterial cell suspension. The pots were observed for diseases such as a yellowing of the leaves after 40 days with a 20-day interval, and root rot and darkening of vascular tissues were observed after 40 days after sowing [88]. Fusarium wilt disease severity was visually assessed on a 0–5 rating. The severity was calculated using the percentage of foliage with visible yellowing or necrosis along with acropetal progression compared with the control pots. The scale of 0 = healthier plants, 1 = 1 to 20% wilt disease on one fourth leaves, 2 = 21 to 49% wilt disease on two fourth leaves, 3 = 50 to 70% wilt disease on three fourth leaves, 4 = 71 to 100% wilt disease on the whole plant and 5 = all leaves dead [89]. The percentage of disease incidence (DI) [90], disease severity (DS), and disease reduction (DR) [89] was scored 40 days (with 20 days interval) after sowing according to the formulas below:

(4) DI (%)=(No. of attained leaves/Total no. of leaves)×100 

(5) DS (%)=(Σ n × v/N × V)×100

where *n* = number of leaves infected by wilt, v = scale of disease severity (0 to 5), N = number of leaves observed, V = value the highest scale [89].

(6) DR (%)=[1−(Disease Incidence in treatment/Disease incidence in control)]×100 

Plant growth parameters, i.e., root and shoot length, fresh and dry weight of root and shoot, and the seed vigor index was measured for each plant after the completion of the experiments.

### 2.8. Detection of Antifungal Antibiotic Genes Present in A7 by PCR Amplification

The PCR-based detection of biomarker genes for the biosynthesis of antifungal antibiotics was achieved using gene-specific primer sets and cycling conditions listed in Table 1. Primers were used for the detection of gene *phlD* for 2,4-diacetylphloroglucinol (2,4-DAPG) [91], *hcnBC* for hydrogen cyanide (HCN) [92], *phzC*, and *phzD* for phenazine-1-carboxylic acid (PCA) [91], *phzH* for phenazine-1-carboxamide (PCN) [93], *prnD* for pyrrolnitrin (PRN) and *pltC* for pyoluteorin (PLT) [94] and *darS* or *darR* for 2-hexyl-5-propyl resorcinol (DarA, DarB enzymes site removed) [95] (Table 1). For each gene, a 10 µL PCR reaction consisted of 0.5 µM primers (forward and reverse), 10 ng A7 genomic DNA, 10× PCR buffer, 200 µM each dNTP, and 0.1U *Taq* DNA polymerase (Qiagen, Hilden, Germany).

### 2.9. Characterization and Analysis of Bioactive Natural Compounds Extract Present in A7 by TLC, HPLC, and GC-MS

To determine the bioactive natural compounds responsible for the antifungal and antibacterial activities of A7, the strain was cultivated in 1 L KB broth and grown for 5 days at 30 ± 2 °C with shaking at 150 rpm [96]. Cells were centrifuged at 8000 rpm for 10 min, and the resulting cell-free supernatant (CFS) was acidified to pH-2 by adding concentrated hydrochloric acid. The acidified CFS was mixed with equal volume (1:1 *v*/*v*) of ethyl acetate and incubated until the separation of two layers. The organic layer was decanted and evaporated at room temperature. The dry residue obtained was dissolved in 250 µL of methanol. An amount of 10 µL was evaluated for antifungal activity along with the control (10 µL ddH_2_O) by the agar-disc diffusion method described earlier. Aliquots of extracts were filtered and analyzed by Thin-layer chromatography (TLC) [97] and High-Performance Liquid Chromatography (HPLC). TLC using silica-G plates activated at 110 °C for 30 min, were used to fractionate the extracted organic compound in chloroform/acetone (9:1 *v*/*v*). The chromatogram was visible under UV light (Bio Red) at 254 nm after the TLC plate had dried [98]. The R_f_ value was calculated after the separation of the fractions, marked, recovered again in methanol, and evaluated again for antifungal activity. The maximum antifungal activity showed by the fraction was analyzed by an HPLC system (1260 Infinity, Agilent Technologies, Santa Clara, CA, USA, with a liquid autosampler, at Indian Institute of Technology Guwahati, IITG). The mobile phase was a 45% CH_3_CN solution containing 0.1% H_3_PO_4_, with a speed of 1.0 mL/min. The wavelength detection was fixed at 270 nm [99]. HPLC elutes of the purified extract were examined via Gas Chromatography-Mass Spectroscopy (GC-MS) (Clarus 680 GC and Clarus 600C MS, PerkinElmer, Waltham, MA, USA; Liquid Autosampler, Library Software: Turbomass NIST 2008, at IITG) equipped with Capillary column: Elite-5MS Capillary Column: Length: 60 m, I.D:0.25 mm (Max. Program Temperature 350 °C), Phase Reference: 5% di-phenyl 95% dimethylpolysiloxane (low bleed) and a mass detector activated in EMV mode. The flow rate of the carrier gas (helium) was set at 1.0 mL min^−1^, the injection-port temperature was kept at 250 °C. The column oven was kept at 80 °C for 2 min, followed by 10 °C min^−1^ to 250 °C for 0 min, then at 50 °C min^−1^ to 280 °C for 9 min. Between *m*/*z* 40 and 450, electron impact spectra in a positive ionization mode was obtained [100].

### 2.10. Statistical Analysis

The results are expressed as mean ± standard error mean (SEM) from data collected across experimental reports (*n* = 3 per data point, a biological triplicate, and repeated experiment). Data were analyzed using an analysis of variance (ANOVA), two-way ANOVA with Bonferroni posttests, and one-way ANOVA with Tukey’s multiple comparisons means test to identify a significant difference with *p*-value < 0.05. All statistical analyses were carried out using GraphPad Prism 5 software version 5.01 GraphPad Software Inc., San Diego, CA, USA.

## 3. Results

### 3.1. Isolation of the Strain and Its Morphological and Biochemical Characterization

A total of 24 distinct bacterial strains were obtained from the rhizoids of the gametophyte of *Polytrichum* sp. on King’s Base agar medium supplemented with 100 µM of AlCl_3_. Among these isolates, A7 was selected based on its promising *In vitro* preliminary plant-growth-promoting, biocontrol activities against different plant pathogenic microbes, and other activities. Morphologically, A7 was rod shaped, smooth and green-pigmented. The green-pigmented, fluorescent activity of the strain was identified under UV light after 24 h of inoculation on KB agar medium. The strain reacted negatively but Gram-staining was found positive for the utilization of different carbohydrate sources, namely arabinose, xylose, glucose, trehalose, and melibiose but could not use adonitol, rhamnose, cellobiose, saccharose, raffinose, and lactose. Furthermore, A7 was also positive for the use of malonate, lysine decarboxylation, nitrate reduction, citrate and negative for ONPG, ornithine reduction, phenylalanine deamination, H_2_S production, Voges Proskauers, methyl red, and esculin hydrolysis (Table 2).

### 3.2. A7 Was Identified as Pseudomonas Aeruginosa Using Molecular Tools

**Figure 1:**
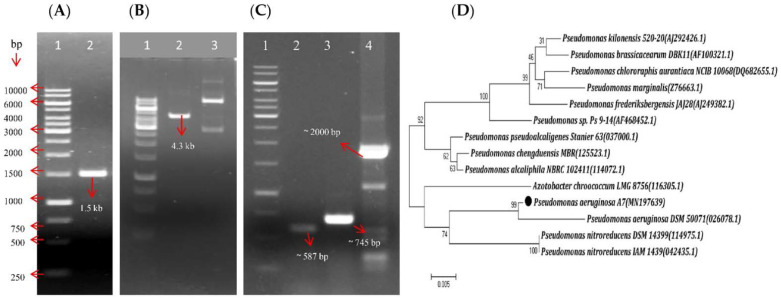
Molecular characterization of the bacterial strain A7. (**A**) Gel image of PCR amplified gene fragment [(lane 1 = Gene Ruler: 1 kb DNA Ladder; lane 2 = 16S rRNA gene fragment with 1.5 kb (kilo base)], (**B**) Gel image of *EcoRI* digested recombinant plasmid DNA fragment (lane 1 = Gene Ruler: 1kb DNA Ladder; lane 2 = digested 4.3 kb recombinant plasmid DNA fragment and lane 3 = undigested recombinant plasmid DNA, (**C**) Gel image of PCR amplified antifungal antibiotics gene fragments (lane 1 = Gene Ruler: 1 kb DNA Ladder; lane 2 = gene fragment *hcnBC* with approx. 587 bp (base pairs); lane 3 = gene fragment *phlD* with approx. 745 bp, lane 4 = gene fragment *phzH* with approx. 2000 bp) and (**D**) Phylogenetic tree analysis of the strain A7 (*Pseudomonas aeruginosa* A7) by Maximum Likelihood method based on the sequence of the 16S rRNA gene of the bacterial strain. Values are given at branch nodes and are based on 1000 replicates. The scale bar indicates 0.005 substitutions per nucleotide position. The accession numbers are enclosed in parenthesis.

The 16S rRNA gene fragment was amplified through PCR amplification with the universal primer set corresponding to a single band (approx. 1.5 kb) (Figure 1A) detected by gel electrophoresis. The transformation of *E. coli* TOP10 with a ligation mix by electroporation resulted in the appearance of small transparent colonies on Luria Bertani (LB) agar plates. The recombinant plasmid was verified by the colony PCR amplification of approx. 1.5 kb gene fragment. *EcoRI* digestion confirmed the recombinant plasmid of size 4.3 kb (Figure 1B). Molecular characterization of the strain A7 was performed based on 16S rRNA gene sequencing identity using blast searches. NCBI blastn analysis of the fragment exhibited 99% similarity with the 16S rRNA gene sequences of *Pseudomonas aeruginosa* DSM 50071, *Pseudomonas aeruginosa* ATCC 10145, and *Pseudomonas aeruginosa* NBRC 12689. The phylogenetic tree of the strain was constructed with the closest *Pseudomonas aeruginosa* strains using the maximum likelihood method with 1000 bootstrap (Figure 1D). The phylogenetic analysis showed that the A7 sequence clustered with several other *Pseudomonas aeruginosa* with high bootstrap support (Figure 1D). Based on this observation, the strain was named *Pseudomonas aeruginosa* A7 and its 16S rRNA gene sequence that was determined deposited in NCBI GenBank with the accession number MN197639.

### 3.3. A7 Showed Bioactivity against Different Phytopathogens

**Figure 2:**
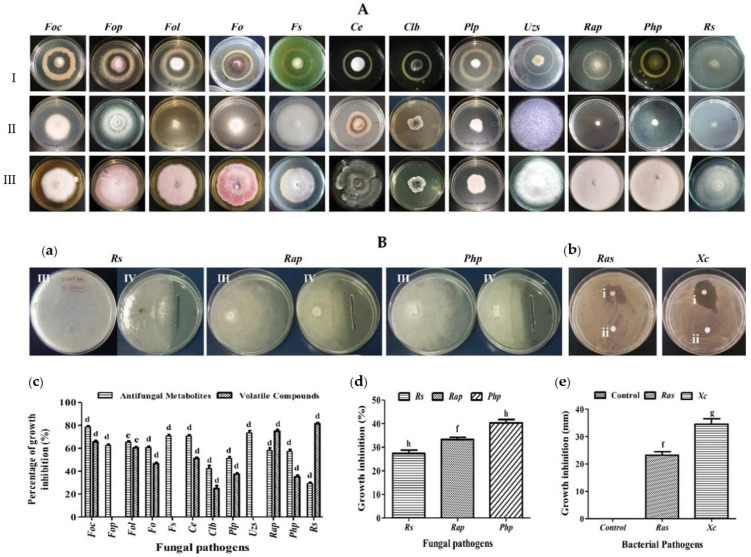
(**A**) A7 bacterial strain inhibits fungal plant pathogens *in vitro*. (I) By concentric ring dual culture bioassay method, (II) By dual plate bioassay method and (III) Control plates of all the fungal pathogens on potato dextrose agar medium at 30 ± 2 °C for a particular incubation period in three biological replicates. Control plates were the same for both experiments, performed for each fungus. *Foc = Fusarium oxysporum* f.sp. *ciceris, Fop = Fusarium oxysporum* f. sp. *pisi, Fol = Fusarium oxysporum* f. sp. *lycopersici* (Sacc.), *Fo = Fusarium oxysporum, Fs = Fusarium solani* (on King’s Base agar medium plate, *Ce = Curvularia eragrostidis, Clb = Curvularia lunata* (Wakker) Boedijn var., *Plp = Phellinus lamaensis* (Murrill) Pat, *Uzs* = *Ustulina zonata* (lev.) Sacc (after seven days of incubation), *Rap* =*Rosellinia arcuata* Petch, *Php* =*Poria hypobrunnea* Petch, and *Rs* =*Rhizoctonia solani* (after two days of incubation). (**B**) *In vitro* antifungal and antibacterial activity of cell-free supernatant (CFS) of A7 against plant fungal and bacterial pathogens. (a) By agar well diffusion method and (b) by agar disk diffusion method on Potato Dextrose Agar and King’s Base agar medium plates at 30 ± 2 °C in three biological replicates. III = Control and IV = Agar well containing 100 µL of CFS on each plate. *Rs* = *Rhizoctonia solani,* *Rap* =*Rosellinia arcuata* Petch and *Php* =*Poria hypobrunnea* Petch. *Ras =* *Ralstonia solanacearum* and *Xc* = *Xanthomonas campestris* (i = agar disc containing 50 µL of CFS and ii = control with 50 µL of sterile double distilled water) after two days of the incubation period. (c) Bar diagram showing the Percentage of growth inhibition by A7 against plant fungal pathogens by producing antifungal metabolites and volatile compounds. Values are expressed as means (*n* = 3) ± Standard Error Mean (SEM). Statistical differences were analyzed using a Two-way analysis of variances (ANOVA). Significant differences (d = *p* < 0.001, e = *p* > 0.05 followed by Bonferroni Posttests) compared with antifungal metabolites vs. volatile compounds. (d) Bar diagram showing the percentage of growth inhibition by the CFS of A7 against plant fungal pathogens and (e) Bar diagram showing growth inhibition of bacterial plant pathogens (calculated as millimeter-scale). Values are expressed as means (*n* = 3) ± Standard Error Mean (SEM). Statistical differences were analyzed using a One-way analysis of variances (ANOVA). Significant differences (f = *p* < 0.001, g = *p* < 0.01 and h = *p* < 0.05, followed by Tukey’s multiple comparison test) compared with the control.

The strain was tested for antagonistic activity against different phytopathogenic fungi (12) and bacteria (2) using the dual-culture technique. The bacterial strain A7 exhibited significant (*p* < 0.001) antimicrobial activity against phytopathogens by producing secondary metabolites including volatile organic compounds (Figure 2, Table 3). The inhibition percentage for fungal pathogens ranged from 29 ± 0.03% to 82 ± 0.04%. The maximum fungal inhibition by A7 by secreting antifungal metabolite was against *Curvularia lunata* (Wakker) Boedijn var. (*Clb*, 82.53 ± 0.9%), followed by, *Fusarium oxysporum* f. sp. *ciceris* (*Foc*, 78.59 ± 1.1%), *Ustillinia zonata* (Lev.) Sacco (*Uzs*, 73.34 ± 2.0%) and the minimum inhibition were for *Rhizoctonia solani* (*Rs*, 29.20 ± 1.03%). The strain A7 also exhibited a moderate growth inhibition of *Rosellinia arcuata* Petch (*Rap*, 57.95 ± 2.3%), *Poriahypobrunnea* Patch (*Php*, 57.26 ± 1.5%), *Phellinus lamaensis* (Murrill) Pat. (*Plp*, 51.01 ± 1.7%), *Fusarium oxysporum* (*Fo*, 60.56 ± 1.2%), *Fusarium oxysporum* f. sp. *lycopersici* (Sacc.) (*Fol*, 65.26 ± 1.2%)*, Fusarium oxysporum* f. sp. *pisi* (*Fop*, 62.51 ± 1.0%), *Fusarium solani* (*Fs*, 70.66 ± 1.2%) and *Curvularia* *eragostidies* (*Ce*, 43.54 ± 0.9% (Figure 2A(a)). The maximum growth inhibition caused by A7 using unidentified volatile organic compounds was against *Rs* (81.25 ± 1.1%), followed by *Rap* (75.99 ± 1.9%), *Clb* (75.95 ± 1.2%), *Foc* (65 ± 0.01%), *Fol* (60.51 ± 1.3%), *Fo* (46.44 ± 1.3%), *Ce* (43.70 ± 1.3%), *Plp* (37.36 ± 1.2%) and *Php* (35.14 ± 1.4%), and was unable to stop the growth of *Clb*, *Fop*, and *Fs* but the morphology of the pathogens altered in these dual cultures (Figure 2A(b),B(c)). The A7′s CFS inhibited the growth of the fungus *Php* (40.33 ± 1.4%), *Rap* (33.33 ± 0.8%), *Rs* (27.33 ± 1.4%). The bacterial pathogens’ growth inhibition measured in diameter was 23.16 ± 1.3 mm for *Ralstonia solanacearum* (*Ras*) and 34.5 ± 2.0 mm for *Xanthomonas campestris* (*Xc*), respectively, compared to the control (sterile de-ionized water) (*p* ˂ 0.05) (Figure 2B(a,b,d,e)) (Table 3).

### 3.4. A7 Is Endowed with Multiple Plant Growth-Promoting Traits

**Figure 3:**
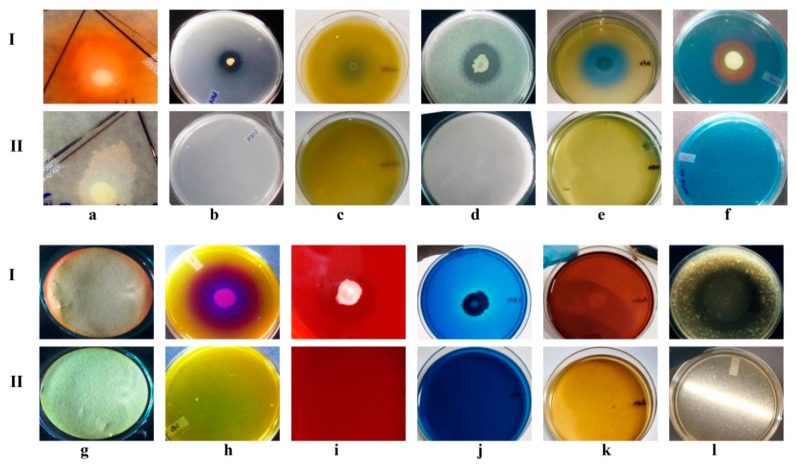
*In vitro* plant-growth-promoting activities of the bacterial strain A7 on different plate-based agar mediums at 30 ± 2 °C for a particular incubation period in three biological replicates. I = Different PGP activities as (**a**) Indole-3-acetic acid production on Whatman no.1 filter paper in Luria Bertani agar (after one day of incubation), (**b**) Phosphate solubilization on Pikovskaya’s agar, (**c**) Potassium solubilization on modified Aleksandrov agar, (**d**) Zinc solubilization on Zinc Oxide agar (after seven days of incubation period), (**e**) Nitrogen fixation on N_2_ free agar (after one day of incubation), (**f**) Siderophores production on CAS agar (after three days of incubation) and (**g**) Hydrogen cyanide production (HCN) on glycine agar (after four days of incubation), (**h**) Chitinase production on colloidal chitin agar (after four days of incubation), (**i**) Cellulase production on Congo—red gelatin agar (after four days of incubation), (**j**) Lignin degradation on Methylene blue indicator MSM—KL agar (after seven days of incubation period), (**k**) Pectinase production on M9 Citrus peel pectin agar (after two days of incubation period), (**l**) Proteases production on Skim milk agar (after seven days of incubation), and II = Control plate of the respective activities.

The A7 plant growth-promoting activities were assessed on different plate-based and liquid-medium-based assays (Figure 3, Table 4). Indole-3-acetic acid production was considered positive after the development of a red halo zone on the Whatman no-1 filter paper membrane around the colony. IAA production was also checked quantitatively with the addition of L-tryptophan at 48 h intervals of incubation. The IAA production increased from 3.3 ± 0.30 to 6.5 ± 0.30 µg/mL during 48 to 96 h of incubation. With a further increase in the incubation time, the production decreased from 5.4 ± 0.03 to 3.1 ± 0.10 µg/mL during 144 to 196 h of incubation (Table 4). The synthesis of IAA without the presence of L-tryptophan was also assessed (Figure S1). The qualitative and quantitative phosphate solubilization activity of A7 was evaluated to be positive, and the solubilization of tri-calcium phosphate was 146 ± 0.50 µg/mL. The A7 was found to be able to solubilize potassium and zinc oxide, and the solubilization efficacy (SE) was recorded at 33% and 29%, respectively (Table 4). The N_2_ fixation was evaluated positively for A7 by the alteration of the color of the medium from pale green to blue (Figure 3e). The diameter of the pale green halo zone was recorded at 52 ± 0.05 mm. The increase in pH related to the creation of ammonia and nitrates from atmospheric N_2_ fixation caused this color shift. Siderophores production was considered positive after the formation of an orange halo around the colony and was measured to be 45 ± 0.05 mm in diameter. Furthermore, the pink appearance in the supernatant indicated the production of siderophores (Figure 3f). The formation of a red-brown tint on filter paper indicated positive HCN synthesis by the strain. Positive ammonia production was also shown by the strain developing brown to yellow precipitate and the strain failed to produce polyamines (data not shown). Chitin degradation was considered positive after the development of the purple zone surrounding the strain from a yellow-colored chitin medium containing Bromocresol purple. In the region of chitin utilization, the pH of the medium shifts from acidic to alkaline due to the production of chitinase enzyme. The diameter of the degraded chitin chain was measured as a purple zone of 58 ± 0.05 mm in diameter. The other enzymatic activity of A7 was measured semi-quantitatively for hydrolytic capability (HC) of different nutrient substrates. The most common enzymatic activity in the bacterial strain A7 was for cellulase (2.4 HC), lignin (+), pectinase (2 HC), and proteases (1.3 HC), (Figure 3i–l and Table 4) and negative for xylanase activity (data not shown).

### 3.5. A7 Is Tolerant to Concentrations of Aluminum and Other Stress Conditions

**Figure 4:**
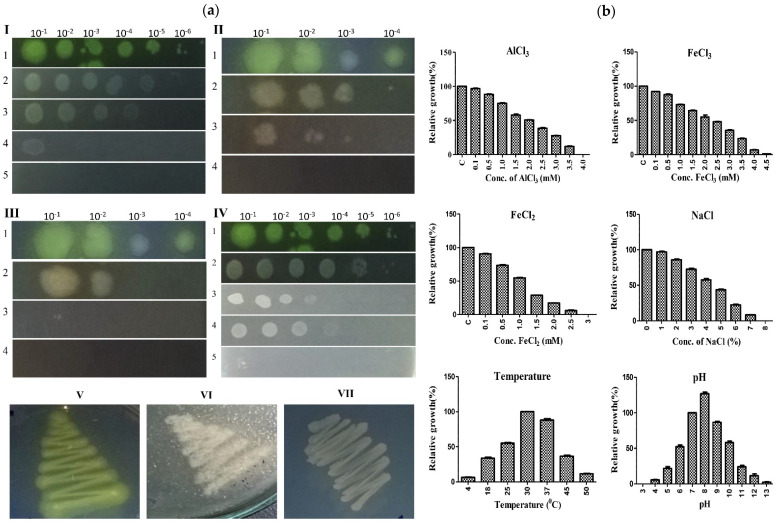
Growth profile of the bacterial strain A7 at different abiotic stress conditions. (**a**) Growth of A7 at different concentrations of metal compounds (FeCl_3_, FeCl_2_, AlCl_3_), pH and temperature by Single Plate Serial Dilution Spotting (SP—SDS) and streaking plate inoculation method at 30 ± 2 °C for a particular incubation period in three biological replicates. (I) AlCl_3_ (1 = control, 2 = 1 mM, 3 = 2 mM, 4 = 3 mM 5 = 4 mM) (at a dilutions of 10^−1^ to 10^−6^), (II) FeCl_3_ (1 = control, 2 = 2 mM, 3 = 4 mM, 4 = 6 mM), (III) FeCl_2_ (1 = control, 2 = 2 mM, 3 = 4 mM, 4 = 6 mM) (at a dilutions of 10^−1^ to 10^−4^), (IV) pH (1 = control, pH—7, 2 = pH—6, 3 = pH—5.5, 4 = pH—5, 5 = pH—4.5) (at a dilutions of 10^−1^ to 10^−6^), (V) Growth of the bacterial strain A7 at 30 °C as control, (VI) Growth of the bacterial strain A7 at 4 °C, (VII) Growth of the bacterial strain A7 at 50 °C after two days of incubation period. (**b**) Bar diagram showing Relative growth of the bacterial strain A7 at different concentrations of metal compounds (FeCl_3_, FeCl_2_, and AlCl_3_), NaCl, Temperature and pH. Values are expressed as means (*n* = 3) ± Standard Error Mean (SEM).

To assess the metal tolerance potential of A7, the strain was tested for its growth on high aluminum and iron amended solid and liquid medium. The A7 could grow and tolerate 0–3.5 mM of aluminum on both the SP-SDS and MM broth medium. The minimum growth inhibitory concentration of A7 for the metal aluminum, i.e., IC_20_ and IC_50_, was 0.76 ± 0.03 and 1.9 ± 0.05 mM. The strain could also tolerate and grow in the presence of FeCl_3_ (up to 4.5 mM) and FeCl_2_ (up to 2.5 mM). The IC_20_ and IC_50_ values were calculated for FeCl_3_ (1.00 ± 0.04, 2.51 ± 0.02 mM) and FeCl_2_ (0.47 ± 0.05, 1.18 ± 0.08 mM). Based on the different concentrations for the three metal compounds, the relative growth was equal, i.e., 73–96% at a concentration of 0.1–1.0 mM for AlCl_3_ and FeCl_3_. Beyond this concentration, the relative growth decreased, and stopped at a concentration of 4, 4.5, and 3 mM for the three metal compounds, respectively. The strain was also checked for temperature, salinity, and pH tolerance potentials. The optimum temperature for A7 growth was 25–40 °C, with diminished growth and reduced fluorescent activity below 10 or above 40 °C (Figure 4a, panel V–VII). The A7 could tolerate salinity stress by up to 7% of NaCl. The optimum pH range for A7 was from pH 6 to 9 with maximum growth at pH-8, as the relative growth was recorded as 126.97 ± 2.0% as compared to control (pH-7). (Figure 4b and Table 5).

### 3.6. In Vitro Protective Effect of A7 against Aluminum Toxicity in Chickpea

**Figure 5:**
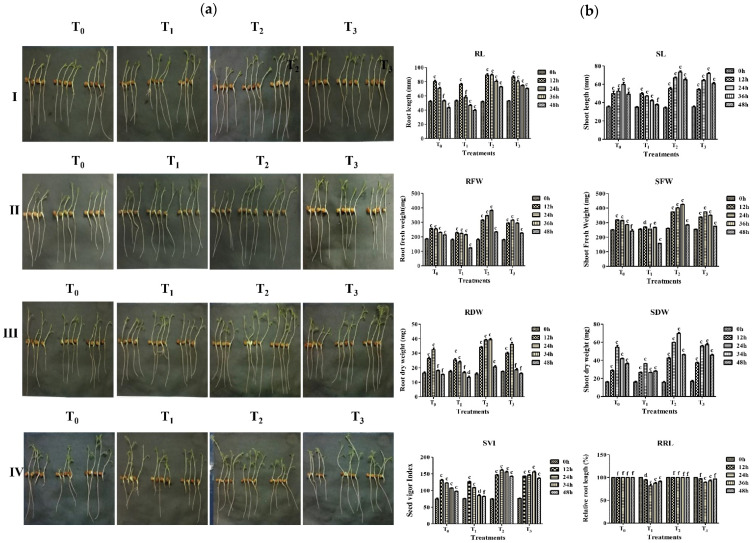
(**a**) Influence of the bacterial strain A7 against aluminum toxicity on the growth promotion of chickpea at 12 h interval of time for 48 h at 14 h photoperiod in white light with a photon flux intensity of 220 mol m^−2^ s^1^ (PAR) in three biological replicates. (I) at 12 h, (II) at 24 h, (III) at 36 h, and (IV) at 48 h. T_0_ = Control (with no bacteria and aluminum), T_1_ = AlCl_3_ (only 100 µM of aluminum chloride), T_2_ = A7 (only freshly grown 0.1 OD_600_ cells of A7) and (4) T_3_ = A7 + AlCl_3_ (freshly grown 0.1 OD_600_ cells of A7 + 100 µM of aluminum chloride). (**b**) Bar diagram showing different growth parameters of the chickpea plants with the influence of the bacterial strain A7 against aluminum toxicity after 12, 24, 36, and 48 h of incubation. RL = Root Length, SL = Shoot Length, RFW = Root Fresh Weight, SFW = Shoot Fresh Weight, RDW = Root Dry Weight, SDW = Shoot Dry Weight, SVI = Seed Vigor Index and RRL = Root Relative Length. Values are expressed as means (*n* = 3) ± Standard Error Mean (SEM). Each replicate consists of sixteen plants per jar. Statistical differences were analyzed by two-way analysis of variances (ANOVA). Significant differences (c = *p* < 0.001, d = *p* < 0.01, e = *p* < 0.05 and f = *p* > 0.05, followed by Bonferroni Posttests) compared with the control.

Since strain A7 was isolated on an aluminum enriched medium, it was further tested for its ability to grow chickpea seedlings in aluminum conditions. The impact of the four treatments on the overall growth of chickpea, i.e., the root, shoot length, root, shoot fresh and dry weight, relative root length rate, was observed with 12 h time intervals for 48 h. The treatment of chickpea seedling by strain A7 in the presence of 100 μM FeCl_3_ solution resulted in a significant (*p* ˂ 0.001) increase in root length, shoot length, root-shoot fresh and dry biomass, seedling vigor index, and the relative root-length rate compared to only aluminum treated seedlings (Figure 5a,b). When the seedlings were treated only with aluminum, morphological changes on the root and root-growth inhibition were observed. When the seedlings were treated with A7 in presence of aluminum, the root length, as well as the fresh and dry weight of the root, exhibited a similar trend to the treatment of seedlings with A7 alone. The protective effect of A7 on aluminum treated chickpea was measured in terms of root length (max. 89 ± 1.4 mm and min. 42 ± 1.7 mm) and root fresh (max. 382 ± 3.8 mg and min. 123 ± 1.7 mg) root dry weight (max. 40 ± 0.8 mg and min. 13 ± 0.8 mg) relative root length rate (max. 97 ± 0.3% and min 82 ± 3.0%). The values of seed vigor index, i.e., plant overall growth was recorded as max. 1530 ± 3.0 and min. 1345 ± 1.5, respectively, during the incubation period (Figure 5 and Table 6).

### 3.7. A7 Was a Potential Biocontrol Candidate against Fusarium Wilt of Chickpea

**Figure 6:**
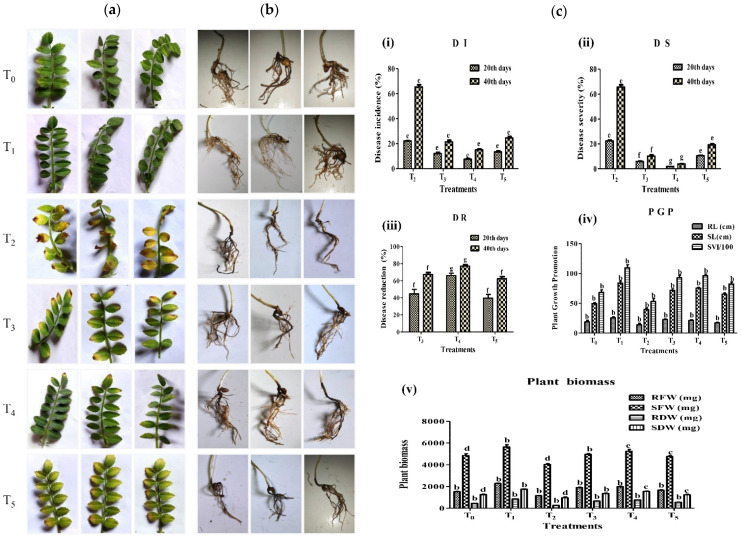
*In vivo* establishment and reduction of chickpea wilt caused by *Fusarium oxysporum* f. sp. *ciceris* by the bacterial strain A7. The data in panels a and b is after 40th days of inoculation with the treatments as T_0_ = Sterile distilled water as control, T_1_ = Only bacterial cell suspension, T_2_ = Only fungal spore suspension, T_3_ = Bacterial cell suspension and fungal spore suspension, T_4_ = Bacterial cell suspension two days before the fungal spore suspension and T_5_ = Fungal spore suspension two days before the bacterial cell suspension at room temperature in three biological replicates. (**a**) Yellowing of leaves, (**b**) Root rot, and darkening of vascular tissues. (**c**) Bar diagram showing the percentage of (i) disease incidence (DI), (ii) disease severity (DS), and (iii) disease reduction (DR) of Fusarium wilt of chickpea from pathogen *Fusarium oxysporum* f. sp. *ciceris* by the strain A7 after 20th and 40th days of inoculation. Values are expressed as means (*n* = 3) ± Standard Error Mean (SEM). Statistical differences were analyzed by a Two-way analysis of variances (ANOVA). Significant differences (e = *p* < 0.001, f = *p* < 0.01 and g = *p* > 0.05, followed by Bonferroni Posttests) compared with the control. Bar diagram also shows (iv) plant-growth promotion (PGP) and (v) plant biomass of chickpea during the establishment of Fusarium wilt of chickpea caused by *Fusarium oxysporum* f. sp. *ciceris* after 40 days of inoculation. Values are expressed as means (*n* = 3) ± Standard Error Mean (SEM). Statistical differences were analyzed by two-way analysis of variances (ANOVA). Significant differences (b = *p* < 0.001, c = *p* < 0.01 and d = *p* > 0.05, followed by Bonferroni Posttests) compared with the control.

The *in vivo* control of *Foc* wilt disease of chickpea by strain A7 was evaluated in pot experiments using six treatments in which three treatments (T_3_–T_5_) involved A7 and *Foc*. The disease symptoms on the leaves of chickpea such as yellowing or necrosis from top to bottom were observed and recorded after the 20th and 40th days after inoculation while root rot was observed after 40th days of inoculation (Figure 6 and Figure S2A–C and Table 7). These symptoms led to the drying of leaves, drooping of petioles, and darkening of vascular tissues which are characteristic symptoms of *Foc* wilting. In comparison to the pathogen-inoculated positive control, all treatments, including A7, consistently suppressed the disease. When the chickpea seedlings were treated only with the spore suspension of *Foc*, severe wilt disease developed on the plants, and the percentage of disease incidence was 22.03 ± 0.4% to 65.43 ± 1.7%, and the disease severity was 22.43 ± 0.8% to 65.54 ± 1.7% (Table 7). The percentage of disease incidence was 12.12 ± 1.0 to 21.37 ± 1.3%, disease severity was 5.95 ± 0.5 to 10.29 ± 0.8%, and disease reduction was 44.70 ± 5.2 to 67.23 ± 2.3% when both A7 and *Foc* were inoculated in the soil simultaneously. Interestingly, when the soil was first treated with A7 before two days of the inoculation of *Foc*, the disease reduction was maximum 66.01 ± 2.9 to 77.00 ± 1.5% and minimum disease incidence 7.4 ± 0.6 to 14.99 ± 0.6 and disease severity 1.85 ± 0.1% to 3.92 ± 0.1% was observed from the plants compared to the soil treated only with *Foc*. Similarly, when the soil was treated with *Foc* prior two days to the inoculation of A7, the disease incidence was 13.20 ± 1.0 to 24.56 ± 1.0%, disease severity was 10.48 ± 0.5% to 19.38 ± 0.6%, and the disease reduction was 39.73 ± 1.6 to 62.26 ± 2.5%. From these different combinations of treatments, it was clear that strain A7 showed maximum disease protection when it was applied two days before inoculation of the pathogen and minimum when it was applied two days after the inoculation of the pathogen. From these experimental settings, the plant-growth-promoting activities of strain A7 on chickpea were also observed and recorded. The application of A7 on the plants resulted in the healthy growth of plants compared with the non-inoculated control at the end of 40th days of inoculation. The maximum root length, shoot length and plant biomass was recorded when treated with only A7 compared to control. The PGP parameters including root length, shoot length, root and shoot fresh and dry weight, and the seed vigor index were observed and recorded in different treatments (*p* < 0.05) (Figure 6, Table 8).

### 3.8. A7 Genome Had Antibiotics, DAPG, HCN, and PCN Biosynthesis Capabilities

The A7 genomic DNA was used for PCR amplification and verification of antibiotic marker genes, which suggested the presence of three gene segments encoding for, 2, 4-diacetylphloroglucinol (2, 4-DAPG), hydrogen cyanide production (HCN), and phenazine-1-carboxamide (PCN), respectively (Figure 1D). DNA fragments of sizes of 745 bp of *phlD*, 587 bp of *hcnBC*, and 2000 bp of *phzH* genes were amplified using gene-specific primer sets. These are the major genes engaged in the production of 2, 4-DAPG, HCN, and PCN, respectively. However, *prnD* for pyrrolnitrin (PRN), *phzC* and *phzD* for Phenazine-1-Carboxylic acid (PCA), *pltC* for pyoluteorin, and *darS* or *darR* for 2-hexyl-5-propyl resorcinol (DarA, DarB) genes were not detected by PCR using gene-specific primer sets mentioned in Table 1.

### 3.9. Characterization and Analysis of Bioactive Natural Compounds in A7 through TLC, HPLC, and GC-MS

**Figure 7:**
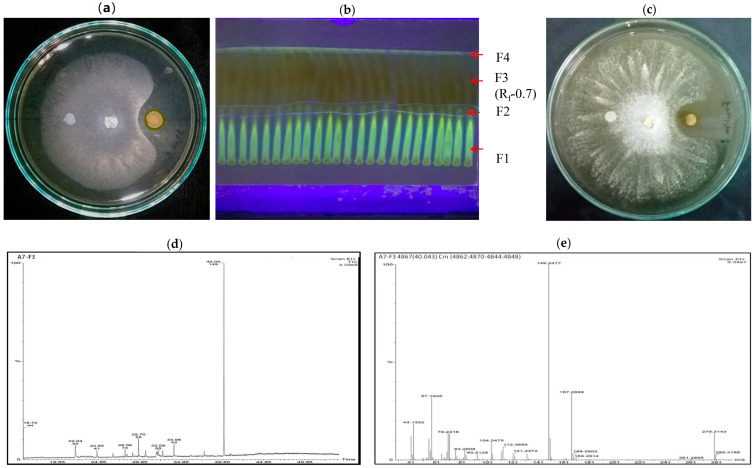
Bio-active compounds produced by the bacterial strain A7 in three biological replicates. (**a**) Antifungal activity of the dried ethyl acetate extract of the strain A7 (before thin layer chromatographic separation) against *Rosellinia arcuata* Patch. (**b**) TLC plate exhibited four fractions of the dried ethyl acetate extract of the bacterial strain with a single discrete fraction (F_3_) with 0.70 R_f_ value, (**c**) Antifungal activity of the methanol extract of TLC purified fraction F_3_ against *Rosellinia arcuata* Patch, (**d**) Gas Chromatography (GC) analysis of the TLC purified fraction F_3_ of A7 for compound characterization and (**e**) Mass spectra (MS) analysis of the fraction F_3_ of A7 for compound identification.

The extracted methanol organic mixture from A7 showed a 35% growth inhibition of *Rosellinia arcuata* Petch compared to control. Four main fractions, namely, F_1_, F_2_, F_3_, and F_4_, were exhibited on the TLC plate when observed under UV light (Bio Red) at 254 nm. The fraction F_3_ was further purified, and a single discrete spot was obtained with a 0.70R_f_ value. The final purified methanol extract showed 30% growth inhibition of Rap (Figure 7). The compound’s retention time was 6.080 min, according to HPLC analysis (data not shown). Therefore, the partially purified fraction was selected for GC-MS for further characterization. The National Institute of Standards and Technology (NIST) library identified many chemical compounds. Among them, twenty-two compounds were selected dependent on their molecular formula, molecular mass, and retention time (RT). These compounds are Oleic acid; 4-fluoro-1-methyl-5-carboxylic acid; 2-tridecenoic acid and 6,10-dimethyl-4-undecanol (RT 22.04), 1-pentadecene; 1-tridecene; 10-heneicosene (C, T) and 1-hexadecene (RT 24.65); Tridecanoic acid; 12-methyl-ester; Hexadecanoic acid; methyl ester; Tridecanoic acid, methyl ester; Hexacosanoic acid methyl ester (RT-28.06); 5-eicosene; E-15-heptadecena; Cycloeicosane; and 1-heptadecene (RT 29.70), 9-octadecenoic acid; 12-octadecenoic acid; Trans-13-octadecenoic acid (RT 32.80); 1,2-benzenedicarboxylic acid; mono (2-ethylhexyl) ester; 6-ethyloct-3-yl-2-ethylhexyl ester; and 1,2-benzenedicarboxylic acid, Diisooctyl ester, Bis (2-ethylhexyl) phthalate (RT 40.04) (Table 9).

## 4. Discussion

In the current research, based on its 16S rRNA sequences and phylogenetic analyses, aluminum-tolerant A7 obtained from the rhizoids of *Polytrichum* sp. was identified as *Pseudomonas aeruginosa*. Morphologically, A7 was Gram-negative, coccus, round, smooth and green pigmented [105]. In this study, multiple phenotypes of A7 related to plant growth promotion (PGP) and biocontrol potentials are reported. The biological control of plant diseases using bacteria has evolved as a sustainable disease-management approach. Bacteria associated with the root or in the rhizosphere are capable of secreting a range of antibiotic compounds, making them a potential source of biocontrol agents [106]. *Burkholderia* sp., *Pseudomonas* sp., *Flavobacterium* sp., and *Bacillus* sp. are examples of antifungal and PGP root and rhizobacteria. They play an important role in plant-growth promotion by secreting phytohormones, most importantly Indole-3-acetic acid (IAA) [107,108]. The inability of *P. putida* GR12-2 deficient in IAA synthesis to increase root growth and lateral-root formation was indicative of the role of bacterial IAA in plant benefits [109]. Soil fertility and plant nutrition are critical for ensuring adequate levels of micronutrients such as potassium, phosphorus, zinc, and many others, necessary for plant growth and development [110]. A PGPR that solubilizes phosphate has great potential in agricultural applications. Richardson et al. [111] found that bacteria increased the amount of P available in the soil for plant uptake. Similarly, in this study, *Pseudomonas aeruginosa* A7 could solubilize tri-calcium phosphate *in vitro*. *Pseudomonas* sp. AF-54 showed the possibility of inorganic P-solubilization by reducing pH and releasing acetic, malic, and gluconic acid [112]. The rhizosphere typically contains P-solubilizing bacteria, which produce gluconic acids to liberate inorganic phosphate from the soil [113,114]. The action of PGPR is frequently linked to the regulation of phytohormones involved in root development and growth as well as phosphorus solubilization [115]. Potassium-solubilizing bacteria have been observed to produce potassium from decomposing organic matter, crop residues, aluminosilicates, kaolinites, and feldspars by acidosis, chelation, and complex formation and exchange reactions [65,116,117,118]. *Bacillus* sp., *Burkholderia* sp., and *Pseudomonas* sp. have been reported to efficiently solubilize potassium from K-feldspar from tea-planted soil [119]. According to [67,120] *Rahnella aquatilis, Pseudomonas orientalis*, *Pantoea agglomerans*, and *Enterobacter* isolated from the rhizosphere of paddy soil and rubber plant-soil, respectively, may solubilize potassium. *Arthrobacter* sp., *Mesorhizobium* sp., *Paenibacillus* sp., *Klebsiella variicola* and *Enterobacter cloacae* rape rhizobacteria and *Klebsiella variicola*, and *Enterobacter cloacae* tobacco rhizobacteria are reported to be able to solubilize potassium [121,122]. To reduce the use of chemicals, nitrogen fertilizer application in plants and atmospheric nitrogen fixation in soil by bacterial strains boost nitrogen availability in soil vital for plant growth [110]. Rhizospheres such as *Aster gymnocephalus*, *Gringelia* sp., *Haplopappus* sp., *Juniperus* sp. and *Lygodesmia* sp., are the major source of the nitrogen-fixing genus *Paenibacillus.* However, *Azospirillum lipoferum* and *Bradyrhizobium japonicum* were the reported rhizobacteria of *Haplopappus* sp., and *Viguiera linearis* [123]. It has also been suggested that root-associated *Pseudomonas stutzeri* A1501 fixes atmospheric nitrogen [124]. Diazotrophs, isolated from pepper vines, possess an enzyme that can convert N_2_ into NH_4_^+^ that plants can take up through the biological nitrogen fixation process [125,126]. *Azospirillum brasilense* sp. 245 and *Burkholderia kururiensis* M130 improved lateral-root development and ethylene signaling responses during rice-seedling cultivation through the biological nitrogen fixation process [127]. Interestingly, in this study, A7 exhibited nitrogen fixation in the nitrogen-free medium, which can be explored for the mechanism and future use. *Agrobacterium tumefaciens*, *Rhizobium* sp. [67]. *Pseudomonas* and *Gluconacetobacter* are capable of oxidizing zinc in a solid environment [68]. PGPR, including *Pseudomonas aeruginosa* A7, produces iron-chelating agents that bind insoluble ferric ions, thereby inhibiting the growth of phytopathogens and thus helping to control plant diseases. Siderophores are metal-chelating agents of low molecular weight that produced by bacteria and secreted as a means of facilitating iron uptake into their cells under Fe-limiting conditions [71,128]. Moreover, siderophores can bind a variety of metals, in addition to iron and several chemical structures and properties. The bacterial siderophores act as biocontrol agents and help in plant growth, thus applicable in mineral weathering, biosensors, bioremediation, and chelation agents [129]. Various physiologic and biochemical activities in plant growth and metabolism involve either root-based chelate breakdown or ligand exchange to provide iron and other metals from bound siderophores [130,131].

Multiple rhizo-*Pseudomonas* from diverse environments are known to decrease plant diseases through the secretion of natural product metabolites [105,132]. Extracellular enzyme-like cellulase, chitinase, and proteases produced by these bacteria attack the cell wall of plant pathogenic fungi, causing cell lysis and subsequent death [133]. Chitinase hydrolyzes the polysaccharide chitin, a linear homopolymer of β-1,4-linked *N*-acetyl-d-glucosamine (GlcNAc) residue found in the cell wall of fungi [134,135]. Antifungal protein degrading enzymes, Proteases, are known to be produced by biocontrol and PGP bacteria [136]. Several species of *Arthrobacter*, *Pseudomonas, Sphingobium, Streptomyces, and Rhodococcus* possess ligninolytic activity *in vitro* [137]. Fungal pathogens and PGPR enzymes, the most effective agents of biological control of plant diseases caused by fungal pathogens hydrolyze the 1,4-glycosidic bonds between the *N*-acetyl glucosamine in fungal chitin [73]. *Pseudomonas aeruginosa* A7 was found to degrade chitin and demonstrated wide spectrum fungal growth inhibition suggesting one such biocontrol mechanism. Hydrogen cyanide (HCN), a major inhibitor of cytochrome c oxidase and several other metalloenzymes, is produced and secreted by a few bacterial species as a secondary metabolite [138]. HCN synthase is a membrane-bound flavoenzyme present in proteobacteria (especially *Fluorescent pseudomonads*) that oxidizes glycine to generate HCN and CO_2_. Sequence similarities exist between the *hcnABC* structural genes of *Pseudomonas fluorescens* and *Pseudomonas aeruginosa* and genes encoding different amino acid dehydrogenases/oxidases, particularly nopaline oxidase of *Agrobacterium tumefaciens* [138]. PGPB produces hydrogen cyanide that interferes with essential enzymes leading to the degradation of phytopathogens cell walls and their control in rhizosphere soil [139,140]. HCN has been associated with the control of *M. javanica* disease in tomatoes and *O. obesity*, an agricultural pest in India [141,142]. Both NIA-2 and NIA-5, as well as *Pseudomonas fluorescence,* impart a beneficial effect on wheat and banana growth in studies [143,144].

The use of bacteria that promotes plant growth in metal-rich environments has been touted as one of the newest trends since metal-tolerant bacteria can support the diverse growth of crops in metal-rich environments [28]. Al can have a beneficial or toxic effect on plants and other organisms, depending on factors such as metal concentration, chemical form, and growth conditions. Al has largely been studied for its physiological, biochemical, and molecular effects on plants (increased nutrient uptake, root growth stimulation, and enzyme activity stimulation, among other things) [145]. Plant growth is adversely affected by metal in acid soil, characterized by a deficiency of nutrients and toxicity by aluminum is the main limiting factor [146]. It has been shown that aluminum inhibits the rapid expansion of roots in plants and the rate of relative root length provides a standard indicator of the toxicity of aluminum [36]. It was shown that metal-tolerant bacteria can advance plant development and increase metal uptake by hyper-and non-hyperaccumulator plants [147]. *Bacillus* sp. KGMDI and *Bacillus* sp. K25 tolerate their growth up to 75 mg/L, equal to 0.56 mM of aluminum, iron, and copper [148]. A7 could tolerate AlCl_3_ up to 3.5 mM, FeCl_3_ up to 4.5 mM and FeCl_2_ up to 2 mM. In this study, *Pseudomonas aeruginosa* A7 produced IAA in the presence of 100 µM AlCl_3_, which may be responsible for the growth of chickpea by increasing root and shoot elongation, leaf surface area, and dry biomass. *Burkholderia ginsengiterrae* N11-2, *Chryseobacterium polytrichastri* N10, *Pseudomonas fluorescens, Pseudomonas fragi* N8, and *Pseudomonas simiae* N3, have been reported to grow in aluminum stress conditions [28,29].

Several PGPR strains are reported to have been used in the integrated biological control of Fusarium wilt in chickpea [89,149,150]. Fusarium wilt, produced by the soil-borne fungus *Fusarium oxysporum ciceris* is a major issue restricting chickpea output around the world [9,151]. The disease symptoms such as yellowing of leaves, abnormal branching, drooping petioles, necrosis of foliage, and drying of roots are early symptoms [9,152]. Currently, chemical-control approaches have no effect on the Fusarium wilt of chickpea control in the field, so biological control is the most promising and effective method [153]. In this study, we demonstrated that strain A7 showed antagonistic activities against *Foc* under *in vitro* and *in vivo* conditions. Remarkably, the strain was able to promote chickpea growth and no negative impact on its growth was observed. PGPR genus *Bacillus* (63.14%), *Burkholderia*, and *Pseudomonas* (53.52%) have been reported to be effectively exploited as control agents for the pathogen by producing HCN pyrrolnitrin, phloroglucinol, phenazine, siderophores, cell-wall-degradation enzymes that suppress the *Fusarium oxysporum* f. sp. *ciceris* by inhibiting mycelial growth. As a result, they play a critical role in a variety of microbial interactions, resulting in abnormal hyphal swelling, becoming exceptionally lengthy and vacuolization, which manipulates physiological processes in other fungi and bacteria [26,150,154,155]. In our *in vivo* experiment, we found that the PGPR strain A7 consistently suppressed yellowing disease caused by *Foc* when applied to the soil. The A7 strain significantly reduced the yellowing disease and root rot in chickpea plants compared to the pathogen-inoculated control. So, this strain could be an excellent choice for further biological control research and chickpea-farming success.

The presence of antifungal antibiotic genes in the bacterial strain A7 was confirmed by a polymerase chain reaction for marker genes for the synthesis of 2,4-DAPG, hydrogen cyanide synthase, and phenazine-1-carboxamide. These antibiotics are known to target plasma-membrane integrity or electron transport in many fungal and bacterial pathogens and indirectly help plant growth [156,157,158]. Additionally, A7 was shown to produce several interesting bioactive compounds with known and novel activities. According to earlier research, lipid derivatives such as 9-octadecanoic acid, hexadecanoic acid methyl ester, octadecenoic acid, 1,2-benzene dicarboxylic acid, mono (2-Ethylhexyl) ester, 1,2-benzene dicarboxylic acid and diisooctyl ester [159] were found to be powerful antibacterial compounds [103]. They induced the autolysis of cell membranes, damaged cell walls, and inhibited transcription, and translation in *S. aureus*, *E. coli*, and *Salmonella* sp. [160]. Interestingly these compounds have been reported to have antimicrobial, anti-inflammatory, antioxidant, and anticancer properties [161]. The antimicrobial and antioxidant activity of 1-hexadecene and hexadecane extracted from *Acanthophora spicifera* was also reported [162]. It is reported that various indirect and direct mechanisms contribute to pseudomonas promoting plant development [163]. Similarly, in this study, we report multiple phenotypes and mechanisms of biocontrol and plant-growth promotion by the reported strain.

In our report, several isolates of *Pseudomonas aeruginosa* have been reported as plant growth-promoting rhizobacteria (PGPR). *Pseudomonas aeruginosa* RRALC3 isolated from degraded forest soil from the Indian state of Chennai, enhanced the growth, macro-micronutrient contents, along with enhanced carbon content of the native legume *Pongamia pinnata* seedlings [164]. *Pseudomonas aeruginosa* isolated from Korean mine soil was reported to have several plant-protection traits including antagonism against bacterial phytopathogens and the stimulation of defense-related genes in tomato plants [165]. PGPR *Pseudomonas aeruginosa* was reported to be the dominant bacteria from the rhizosphere of sorghum, paddy, tobacco, tea, and maize crops in India [166]. Recently, the *Pseudomonas aeruginosa* strain RTE4, isolated from the tea plant rhizosphere [167]; tomato plant rhizosphere associated *Pseudomonas aeruginosa* strain CQ-40 [168] and *Pseudomonas aeruginosa* FG106 [169]; and saffron rhizosphere *Pseudomonas aeruginosa* strain YY322 [170], have been reported as PGPR with multiple beneficial phenotypes and biocontrol activities in the plants.

Using culture-dependent and independent methods, it is suggested that many human pathogenic bacteria from the genus *Burkholderia*, *Enterobacter*, *Pseudomonas*, *Ralstonia*, *Serratia*, *Staphylococcus*, and *Stenotrophomonas* are prominent members of several rhizospheres [171,172]. Several species from these genera possess growth-promoting properties for plants, as well as excellent antagonistic properties against plant pathogens; as a result, they are used to prevent disease and promote plant growth [171]. To use rhizosphere, isolate as a successful biocontrol agent, the strains should be tested for safety and risk assessments should be performed [20]. Human isolates of *Pseudomonas aeruginosa* are recognized as an opportunistic, nosocomial human pathogen and thus the environmental isolates must be extensively researched before being considered as a biological control agent. Thus, the comparative virulence attributes of the reported *Pseudomonas aeruginosa* A7 will allow it to be used as a BCA and a safe biofertilizer. Additionally, since the strain is already adapted to local environmental conditions, it may be transformed into a biofertilizer in future research involving different chickpea varieties, environmental conditions, and field trials. Further research is required to fully understand the multiple biocontrol and chickpea plant growth mechanisms of strain A7. This study thus provides another biocontrol arsenal against wilt disease which can be useful in the control of Fusarium wilt in aluminum and metal-rich soils.

## 5. Conclusions

A *Fusarium oxysporum* f. sp. *ciceris (Foc*) antagonist bacterium was identified from the rhizoids of adult moss, identified as *Pseudomonas aeruginosa* A7 on a morphological and molecular basis. The strain produced antimicrobial metabolites with strong antagonistic activities against multiple plant pathogenic fungi and bacteria. It was shown to have significant inhibitory effects on the Fusarium wilt of chickpea and enhanced chickpea growth in a pot experiment. Chickpea seedlings under aluminum stress grew significantly better when treated with A7. Based on the PCR results, the strain carried several biocontrol markers related to the biosynthesis of antibiotics HCN, 2, 4-DAPG, and PCN. Furthermore, A7′s poly-extremophilic and extensive adaptations to metal stress can be useful in the bioremediation of metal-rich soils. The strain showed promising phenotypes with the potential of improving seedling growth and development while reducing losses caused by fungal plant diseases. Thus, the strain can be used in agriculture as a natural alternative to conventional PGPR and fungicides after the comprehensive biological safety assessment using the tools of whole-genome sequencing, transcriptomics, and metabolomics studies. Additional research is required to establish strain A7’s mechanism of plant-growth promotion in chickpea or other plants. Interestingly, the strains’ lignin degradation and production of bioactive compounds can be harnessed using other biotechnological interventions.

## Figures and Tables

**Table 1:** List of Primers used for identification of the bacterial isolates and antibiotic genes.

Literature Name	Primer Sequence	Primer Characters	PCR Condition (°C)	Ref.
fD1	F-5′-GAGTTTGATCCTGGCTCA-3′	16S rDNA	ID 98 °C for 30 s, FD 98 °C for 10 s, An 50 °C for 30 s,	[53]
rP2	R-5′-ACGGCTACCTTGTTACGACTT-3′		Ex 72 °C at 1.30 min, FEx 72 °C for 7 min.	
Phl2a	F-5′-GAGGACGTCGAAGACCACCA-3′	2,4-DAPG	ID 94 °C for 1.30 min, FD 94 °C for 35 s, An 53 °C for 30 s,	[53]
Phl2b	R-5′-ACCGCAGCATCGTGTATGAG-3′		Ex 72 °C for 45 s, FEx 72 °C for 10 min.	
Aca	F-5′-ACTGCCAGGGGCGGATGTGC-3′	HCN	ID 94 °C for 2.30 min, FD 94 °C for 30 s, An 63 °C for 30 s,	[91,92]
Acb	R-5′-ACGATGTGCTCGGCGTAC-3′		Ex 72 °C for 60 s, FEx 72 °C for 10 min.	
PCA2a	F-5′-TTGCCAAGCCTCGCTCCAAC-3′	PCA	ID 94 °C for 2 min, FD 94 °C for 60 s, An 55 °C for 45 s,	[91]
PCA3b	R-5′-CCGCGTTGTTCCTCGTTCAT-3′		Ex 72 °C for 60 s, FEx 72 °C for 10 min.	
PhzH-up	F-5′-CGCACGGATCCTTTCAGAATGTTC-3′	PCN	ID 98 °C for 30 s, FD 98 °C for 10 s, An 55 °C for 30 s,	[93]
PhzH-low	R-5′-GCCACGCCAAGCTTCACGCTCA-3′		Ex 70 °C for 7 min, FEx 70 °C for 10 min.	
PLTC1	F-5′-AACAGATCGCCCCGGTACAGAACG-3′	PLT	ID 95 °C for 2 min, FD 95 °C for 2 min, An 57 °C for 1 min,	[94]
PLTC2	R-5′-AGGCCCGGACACTCAAGAAACTCG-3′		Ex 72 °C for 1 min, FEx 72 °C for 7 min.	
PRND1	F-5′-GGGGCGGGCCGTGGTGATGGA-3′	PRN	ID 95 °C for 2 min, FD 95 °C for 1 min, An 68 °C for 1 min,	[94]
PRND2	R-5′-YCCCGCSGCCTGYCTGGTCTG-3′		Ex 72 °C for 1 min, FEx 72 °C for 7 min.	
darAF	F-5′-ATCGTCAATGATCTCTGGC-3′	HPR,	ID 94 °C for 2 min, FD 94 °C for 1 min, An 50 °C for 1 min,	[95]
darAR	R-5′-TTATCCACTGCCTCTCCC-3′	DARA,	Ex 72 °C for 1 min, FEx 72 °C for 10 min.	
darBF	F-5′-GATACTCAGCGAGCGTGC-3′	HPR,	ID 94 °C for 2 min, FD 94 °C for 1 min, An 50 °C for 1 min,	
darBR	R-5′-CACCAGGTTATGGCGTCAG-3′	DARB	Ex 72 °C for 1 min, FEx 72 °C for 10 min.	

HCN = Hydrogen cyanide synthase (E), PCA = Phenazine-1-carboxylic acid, PCN = Phenazine-1-carboxamide, PLT = Pyoluteorin, PRN = Pyrrolnitrin, HPR = 2-Hexyl,5-propyl resorcinol, DARA = Dialkylresorcinol gene A, DARB = Dialkylresorcinol gene B, ID = Initial Denaturation temperature, FD = Final Denaturation temperature, An = Annealing temperature, Ex = Extension temperature, FEx = Final Extension temperature. Ref. = References.

**Table 2:** Morphological and biochemical characteristics of the strain A7. + Positive, − Negative.

Morphological Characters		Response	
Shape		Round	
Gram reaction		Negative, rod shaped	
Color		Green pigmented	
**Biochemical**		**Carbohydrate utilization**	
ONGP production	−	Arabinose	+
Lysine Utilization	+	Xylose	+
Ornithine utilization	−	Adonitol	−
Urease production	−	Rhamnose	−
Phenylalanine Deamination	−	Cellobiose	−
Nitrate reduction	+	Melibiose	+
H2S production	−	Saccharose	−
Citrate utilization	+	Raffinose	−
Voges Proskauer’s test	−	Trehalose	+
Methyl red test	−	Glucose	+
Indole production	−	Lactose	−
Malonate utilization	+		
Esculin hydrolysis	−		
Oxidase production	+		

**Table 3:** Effect of the bacterial strain A7 on fungal and bacterial plant pathogen growth inhibition.

Fungal Pathogens	PGI (%) ^a^				
Plant Fungal Pathogens	Source of Collection	Diseased Caused	ITCC No	Antifungal Metabolites	Volatile Compounds
*Fusarium oxysporum* f.sp. *ciceris* (*Foc*)	Chick pea	Fusarium wilt	3636	78.59 ± 1.1 ^d^	65.51 ± 1.3 ^d^
*Fusarium oxysporum* f. sp. *pisi* (*Fop*)	Pea	Wilt	4814	62.51 ± 1.0 ^d^	N/D ^c^
*Fusarium oxysporum* f. sp. *lycopersici* (Sacc.) (*Fol*)	Tomato	Wilt	1322	65.26 ± 1.2 ^e^	60.37 ± 1.0 ^e^
*Fusarium oxysporum* (*Fo*)	Maize Stalk	Wilt	7093	60.56 ± 1.2 ^d^	46.44 ± 1.3 ^d^
*Fusarium solani* (*Fs*)	Paddy soil	Wilt	6953	70.66 ± 1.2 ^d^	N/D ^c^
*Rhizoctonia solani* (*Rs*)	Cucumber	Collar rot	AAU	29.20 ± 1.0 ^d^	81.25 ± 1.1 ^d^
*Curvularia eragrostidis* (*Ce*)	Musa	Leaf spot	5540	70.56 ± 1.3 ^d^	51.00 ± 1.3 ^d^
*Curvularia lunata* (Wakker) Boedijn var. (*Clb*)	Rice	Leaf spot	5193	42.24 ± 2.6 ^d^	24.88 ± 2.5 ^d^
*Rosellinia arcuata* Petch (*Rap*)	Tea bush	Root rot	4143	57.95 ± 2.3 ^d^	74.99 ± 1.5 ^d^
*Poria hypobrunnea* Petch (*Php*)	Tea bush	Stem canker	4141	57.26 ± 1.5 ^d^	35.14 ± 1.4 ^d^
*Phellinus lamaensis* (Murrill) Pat.(*Plp*)	Tea bush	Brown root rot	4140	51.01 ± 1.7 ^d^	37.36 ± 1.2 ^d^
*Ustulina zonata* (Lev.) Sacco (*Uzs*)	Tea bush	Charcoal stump rot	4144	73.34 ± 2.0 ^d^	N/D ^c^
**Plant fungal and bacterial pathogens**				**PGI (%) ^b^**	
*Rhizoctonia solani* (*Rs*)	Cucumber	Collar rot	AAU	27.33 ± 1.4 ^h^	
*Rosellinia arcuata* Petch (*Rap*)	Tea bush	Root rot	4143	33.33 ± 0.8 ^f^	
*Poria hypobrunnea* Petch (*Php*)	Tea bush	Stem canker	4141	40.33 ± 1.4 ^h^	
*Ralstonia solanacearum* (*Ras*)	-	Brown rot	AAU	23.17 ± 1.3 mm ^f^	
*Xanthomonas campestris* (*Xc*)	-	Black rot	AAU	34.50 ± 2.0 mm ^g^	

Percentage of growth inhibition (PGI) of *Pseudomonas aeruginosa* A7 against plant fungal pathogens and growth inhibition of bacterial plant pathogens collected from Indian Type Culture Collection (ITCC) and Assam Agricultural University (AAU). ^a^ PGI (%) = (Dc − Dt)/(Dc) ∗ 100 in dual culture. Values are expressed as means (*n* = 3) ± Standard Error Mean (SEM). Statistical differences were analyzed by a two-way analysis of variances (ANOVA). Significant differences (^d^ *p* < 0.001, ^e^ *p* > 0.05 followed by Bonferroni Posttests) compared with antifungal metabolites and volatile compounds are indicated by letters (^d^ and ^e^). ^b^ PGI (%) = (Dc − Dt)/(Dc) ∗ 100 in presence of Cell-Free Supernatant (CFS) of A7 and growth inhibition of bacterial plant pathogens (mm). Values are expressed as means (*n* = 3) ± Standard Error Mean (SEM). Statistical differences were analyzed by One-way analysis of variances (ANOVA). Significant differences (^f^ *p* < 0.001, ^g^ *p* < 0.01, and ^h^ *p* < 0.05 followed by Tukey’s multiple comparison test) compared with the control. ^c^ N/D—No growth inhibition. mm = millimeter scale.

**Table 4:** Plant-growth-promoting activities of the strain A7 and its enzymatic activities.

Direct and Indirect PGP Traits of A7	Response	Indole-3-Acetic ACID Production		
		Time(h)	With Tryptophan (µg/mL)	Without Tryptophan (µg/mL)
Phosphate solubilization	146.00 ± 0.5 µg/mL	48	3.30 ± 0.3 ^d^	1.30 ± 0.3 ^d^
Potassium solubilization	33% (SE) ^a^	96	6.50 ± 0.3 ^c^	3.60 ± 0.1 ^c^
ZnO solubilization	29% (SE) ^a^	144	5.40 ± 0.4 ^c^	2.50 ± 0.0 ^c^
Nitrogen Fixation	52.00 ± 0.05 mm	196	3.10 ± 0.1 ^e^	2.40 ± 0.2 ^e^
Siderophores production	45.00 ± 0.05 mm			
HCN production	+	Quantitative production of Indole-3-acetic acid at 48 h interval incubation period. Statistical differences were analyzed by two-way analysis of variances (ANOVA).		
Chitinase production	58.00 ± 0.05 mm			
Cellulase production	2.4 (HC) ^b^			
Lignin degradation	+			
Pectinase production	2 (HC) ^b^			
Proteases production	1.3 (HC) ^b^			
Polyamine production	-			
Amylase production	+			
Xylanase production	-			
Catalase production	+			

Different plant-growth-promoting and extracellular enzymatic activities with different measurement units. ^a^ SE (Solubilization Efficacy) = (Diameter of A7/Diameter of the halo zone] ∗ 100, ^b^ HC (hydrolysis capacity) = (Ratio of the halo zone diameter to the colony diameter). mm = millimeter. Values are expressed as means *n* = 3 ± Standard Error Mean (SEM). Significant differences (^c^ *p* < 0.001, ^d^ *p* < 0.01 and ^e^ *p* > 0.05, followed by Bonferroni Posttests) compared with and without tryptophan are indicated by letters ^c^, ^d^, and ^e^.

**Table 5:** Relative growth (RG) of the bacterial strain A7 on different abiotic stress conditions.

AlCl_3_		FeCl_3_		FeCl_2_		NaCl		Temperature		pH	
Conc. (mM)	RG (%) ^a^	Conc. (mM)	RG (%) ^a^	Conc. (mM)	RG (%) ^a^	Conc. (%)	RG (%) ^a^	Temp (°C)	RG (%) ^a^	pH	RG (%) ^a^
C	100.00 ± 0.0	C	100.00 ± 0.0	C	100.00 ± 0.0	C	100.00 ± 0.0	4	6.35 ± 0.4	3	0.00 ± 0.0
0.1	96.55 ± 0.7	0.1	92.03 ± 0.4	0.1	90.54 ± 0.5	1	96.98 ± 1.0	18	33.66 ± 1.4	4	5.50 ± 0.5
0.5	88.25 ± 0.8	0.5	87.50 ± 1.0	0.5	73.38 ± 1.1	2	85.93 ± 1.0	25	55.22 ± 1.0	5	21.59 ± 2.4
1.0	75.00 ± 1.1	1.0	73.20 ± 0.3	1.0	54.75 ± 0.6	3	72.53 ± 1.2	30(C)	100.00 ± 0.0	6	52.31 ± 2.3
1.5	57.81 ± 1.6	1.5	64.34 ± 0.8	1.5	28.78 ± 0.1	4	57.44 ± 1.8	37	88.00 ± 2.0	7(C)	100.00 ± 0.0
2.0	50.74 ± 0.3	2.0	55.07 ± 2.7	2.0	17.21 ± 0.3	5	43.51 ± 1.1	45	36.50 ± 1.5	8	126.97 ± 2.0
2.5	38.70 ± 0.8	2.5	48.19 ± 0.4	2.5	6.08 ± 0.7	6	22.33 ± 1.0	50	11.56 ± 0.4	9	86.50 ± 1.5
3.0	27.63 ± 0.4	3.0	35.70 ± 0.8	3	0.00 ± 0.0	7	8.31 ± 0.3			10	58.00 ± 2.0
3.5	11.94 ± 0.7	3.5	23.62 ± 0.6			8	0.00 ± 0.0			11	24.00 ± 2.0
4.0	0.00 ± 0.0	4.0	6.93 ± 0.5							12	11.50 ± 2.5
		4.5	1.36 ± 0.0							13	2.40 ± 0.5
**Inhibitory Concentration (IC) (mM)**											
Metal	IC_20_^b^	IC_50_^c^	Metal	IC_20_^b^	IC_50_^c^	Metal		IC_20_^b^		IC_50_^c^	
AlCl_3_	0.76 ± 0.03	1.9 ± 0.0	FeCl_3_	1.00 ± 0.04	2.50 ± 0.02	FeCl_2_		0.47 ± 0.05		1.18 ± 0.08	

Percentage Relative Growth of the bacterial strain at different concentrations of metal compounds: AlCl_3_, FeCl_3_, FeCl_2_, at different concentrations of NaCl, at different temperature and at different pH range. ^a^ RG (%) = (Optical density the treatments/Optical density of controls) ∗ 100. mM = millimolar. ^b^ IC_20_ = 20% of growth inhibitory concentration of metals, ^c^ IC_50_ = 50% of growth inhibitory concentration of metals. Conc. = Concentrations, Temp. = Temperature, C = control. Values are expressed as means *n* = 3 ± Standard Error Mean (SEM) each biological replicate.

**Table 6:** Influence of strain A7 on the growth of chickpea in a stressed condition.

Time	Treatment	RL (mm)	SL (mm)	RFW (mg)	SFW (mg)	RDW (mg)	SDW (mg)	SVI ^a^	RRL (%) ^b^
12 h	T_0_	80 ± 2.0 ^c^	50 ± 2.9 ^c^	256 ± 3.0 ^c^	307 ± 3.6 ^c^	26 ± 0.8 ^c^	29 ± 0.8 ^c^	1290 ± 1.0 ^c^	100 ± 0.0 ^f^
	T_1_	76 ± 1.5 ^c^	50 ± 1.2 ^c^	227 ± 3.9 ^c^	292 ± 1.2 ^d^	25 ± 0.8 ^c^	27 ± 0.6 ^c^	1240 ± 2.0 ^c^	95 ± 1.0 ^d^
	T_2_	88 ± 1.4 ^c^	55 ± 1.4 ^c^	316 ± 3.4 ^c^	365 ± 2.6 ^c^	34 ± 0.5 ^c^	42 ± 0.8 ^c^	1445 ± 1.5 ^c^	100 ± 0.0 ^f^
	T_3_	86 ± 1.8 ^c^	54 ± 0.8 ^c^	294 ± 2.3 ^c^	342 ± 1.2 ^c^	30 ± 0.5 ^c^	37 ± 0.8 ^c^	1400 ± 1.0 ^c^	97 ± 0.3 ^f^
24 h	T_0_	71 ± 1.4 ^c^	52 ± 2.6 ^c^	254 ± 2.0 ^c^	334 ± 2.6 ^c^	32 ± 1.2 ^c^	54 ± 2.3 ^c^	1210 ± 1.0 ^c^	100 ± 0.0 ^f^
	T_1_	58 ± 2.6 ^f^	47 ± 0.5 ^c^	224 ± 2.0 ^c^	313 ± 1.7 ^f^	24 ± 1.5 ^c^	36 ± 0.3 ^c^	1077 ± 1.7 ^c^	82 ± 3.0 ^c^
	T_2_	89 ± 1.4 ^c^	67 ± 1.0 ^c^	346 ± 3.1 ^c^	431 ± 1.4 ^c^	39 ± 0.5 ^c^	60 ± 0.3 ^c^	1580 ± 2.0 ^c^	100 ± 0.0 ^f^
	T_3_	79 ± 2.3 ^c^	64 ± 0.8 ^c^	315 ± 2.7 ^c^	401 ± 2.4 ^c^	36 ± 3.3 ^c^	57 ± 0.8 ^c^	1445 ± 1.5 ^c^	89 ± 1.1 ^c^
36 h	T_0_	53 ± 1.1 ^f^	60 ± 2.4 ^c^	231 ± 1.7 ^c^	420 ± 1.2 ^c^	18 ± 0.4 ^f^	42 ± 3.3 ^c^	1060 ± 1.0 ^c^	100 ± 0.0 ^f^
	T_1_	47 ± 1.2 ^e^	42 ± 1.0 ^d^	215 ± 2.9 ^c^	242 ± 1.2 ^e^	16 ± 3.2 ^f^	27 ± 3.3 ^c^	825 ± 1.5 ^d^	88 ± 1.8 ^c^
	T_2_	78 ± 3.1^c^	73 ± 1.2^c^	382 ± 3.8 ^c^	565 ± 7.7 ^c^	40 ± 0.8 ^c^	70 ± 1.2 ^c^	1530 ± 1.0 ^c^	100 ± 0.0 ^f^
	T_3_	78 ± 3.1 ^c^	73 ± 1.2 ^c^	381 ± 3.8 ^c^	565 ± 7.7 ^c^	39 ± 0.8 ^f^	70 ± 1.2 ^c^	1530 ± 1.0 ^c^	100 ± 0.0 ^c^
48 h	T_0_	42 ± 1.7 ^c^	49 ± 2.5 ^c^	213 ± 1.5 ^c^	231 ± 1.2 ^f^	15 ± 0.9 ^f^	36 ± 0.3 ^c^	961.± 1.6 ^c^	100 ± 0.0 ^f^
	T_1_	40 ± 1.2 ^c^	38 ± 0.8 ^f^	123 ± 1.7 ^c^	143 ± 1.8 ^c^	13 ± 0.8 ^d^	27 ± 0.0 ^c^	815 ± 0.5 ^f^	91 ± 1.5 ^c^
	T_2_	72 ± 1.4 ^c^	65 ± 1.7 ^c^	234 ± 2.0 ^c^	292 ± 1.4 ^c^	20 ± 0.8 ^c^	46 ± 0.5 ^c^	1400 ± 1.0 ^c^	100 ± 0.0 ^f^
	T_3_	70 ± 0.6 ^c^	61 ± 1.2 ^c^	225 ± 2.9 ^c^	247 ± 4.7 ^c^	16 ± 0.5 ^f^	45 ± 0.8 ^c^	1345 ± 1.5 ^c^	97 ± 0.3 ^f^

Plant-Growth Promotion of chickpea plant in the presence and absence of aluminum chloride with a 12 h interval of time for 48 h. T_0_ = Control, T_1_ = only AlCl_3_, T_2_ = only bacterial strain A7 and T_3_ = Bacterial strain A7 + AlCl_3_, RL = Root Length, SL = Shoot Length, RFW = Root Fresh Weight, SFW = Shoot Fresh Weight, RDW = Root Dry Weight, SDW = Shoot Dry Weight, ^a^ SVI (Seed Vigor Index) = (Shoot length + root length) ∗ seed germination %, ^b^ RRL (Relative Root Length) = (Root length in aluminum stress condition)/(Root length in control conditions) ∗ 100. Values are expressed as means (*n* = 3) ± Standard Error Mean (SEM). Statistical differences were analyzed by two-way analysis of variances (ANOVA). Significant differences (^c^ *p* < 0.001, ^d^ *p* < 0.01, ^e^ *p* < 0.05 and ^f^ *p* > 0.05, followed by Bonferroni Posttests) compared with control.

**Table 7:** *In vivo* disease incidence, severity, and reduction of Fusarium wilt of chickpea by A7.

After 20th Days of Inoculation				After 40th Days of Inoculation		
Treatments	DI (%) ^a^	DS (%) ^b^	DR (%) ^c^	DI (%) ^a^	DS (%) ^b^	DR (%) ^c^
T_2_	22.03 ± 0.4 ^e^	22.43 ± 0.8 ^e^	N/D ^d^	65.43 ± 1.7 ^e^	65.54 ± 1.7 ^e^	N/D ^d^
T_3_	12.12 ± 1.0 ^e^	5.95 ± 0.5 ^f^	44.70 ± 5.2 ^f^	21.37 ± 1.3 ^e^	10.29 ± 0.8 ^f^	67.23 ± 2.3 ^f^
T_4_	7.4 ± 0.6 ^e^	1.85 ± 0.1 ^g^	66.01 ± 2.9 ^g^	14.99 ± 0.6 ^e^	3.92 ± 0.1 ^g^	77.00 ± 1.5 ^g^
T_5_	13.20 ± 1.0 ^e^	10.48 ± 0.5 ^e^	39.73 ± 1.6 ^f^	24.56 ± 1.0 ^e^	19.38 ± 0.6 ^e^	62.26 ± 2.5 ^f^

Establishment of Fusarium wilt Disease incidence (DI), Disease severity (DS), caused by *Fusarium oxysporum* f. sp. *ciceris* and Disease reduction (DR) by the strain A7 after 20th and 40th days of inoculation with the following treatments: T_2_ = only fungal spore suspension, T_3_ = bacterial cell suspension, and fungal spore suspension, T_4_ = bacterial cell suspension two days before the fungal spore suspension and T_5_ = fungal spore suspension two days before the bacterial cell suspension. ^a^ DI (%) = (No. of attained leaves/Total no. of leaves) ∗ 100, ^b^ DS (%) = (Σ n × v/N × V) ∗ 100 and ^c^ DR (%) = [1 − (DI in treatment/DI in control)] ∗ 100 after 20th and 40th days of inoculation. ^d^ N/D = no disease reduction. Values are expressed as means (*n* = 3) ± Standard Error Mean (SEM). Statistical differences were analyzed by a Two-way analysis of variances (ANOVA). Significant differences (^e^ *p* < 0.001, ^f^ *p* < 0.01 and ^g^ *p* > 0.05, followed by Bonferroni Posttests) compared with the control.

**Table 8:** *In vivo* growth promotion of chickpea plant by the strain A7 during the treatments with *Fusarium oxysporum* f. sp. *ciceris*.

Plant Growth Promotion				Plant Biomass			
T	RL (cm)	SL (cm)	SVI/100 ^a^	RFW (mg)	SFW (mg)	RDW (mg)	SDW(mg)
T_0_	18.66 ± 2.6 ^b^	49.33 ± 2.0 ^b^	68.00 ± 4.6 ^b^	1530.00 ± 26.4 ^d^	4840.00 ± 173.8 ^d^	456.00 ± 14.0 ^d^	1263.33 ± 31.7 ^d^
T_1_	25.66 ± 1.7 ^b^	83.66 ± 3.4 ^b^	109.33 ± 5.2 ^b^	2280.00 ± 43.5 ^a^	5633.33 ± 210.7 ^a^	853.33 ± 26.0 ^a^	1756.66 ± 29.6 ^a^
T_2_	14.00 ± 2.3 ^b^	39.33 ± 2.6 ^b^	53.00 ± 4.9 ^b^	1157.00 ± 29.8 ^d^	4030.00 ± 88.8 ^d^	270.00 ± 17.3 ^d^	966.66 ± 88.1 ^d^
T_3_	22.66 ± 1.7 ^b^	71.66 ± 2.6 ^b^	92.66 ± 4.3 ^b^	1903.33 ± 53.6 ^b^	4963.33 ± 68.3 ^b^	665.66 ± 11.0 ^b^	1363.33 ± 31.7 ^b^
T_4_	21.00 ± 1.1 ^b^	75.33 ± 1.4 ^b^	96.33 ± 2.6 ^b^	1984.66 ± 45.1 ^c^	5240.00 ± 183.3 ^c^	762.00 ± 14.7 ^c^	1556.66 ± 29.6 ^c^
T_5_	17.00 ± 1.1 ^b^	65.33 ± 2.0 ^b^	82.33 ± 3.1 ^b^	1663.00 ± 31.6 ^c^	4763.33 ± 87.6 ^c^	555.66 ± 12.8 ^c^	1240.00 ± 50.3 ^c^

Plant growth promotion of chickpea plant during the establishment of Fusarium wilt disease caused by *Fusarium oxysporum* f. sp. *ciceris* after 40th days of inoculation with the treatments: T_0_ = Sterile distilled water as control, T_1_ = Only bacterial cell suspension, T_2_ = Only fungal spore suspension, T_3_ = Bacterial cell suspension and fungal spore suspension, T_4_ = Bacterial cell suspension two days before the fungal spore suspension and T_5_ = Fungal spore suspension two days before the bacterial cell suspension. RL = Root Length, SL = Shoot Length, ^a^ Seed Vigor Index/100 = (Shoot length + root length) ∗ seed germination %, RFW = Root Fresh Weight, SFW = Shoot Fresh Weight, RDW = Root Dry Weight, SDW = Shoot Dry Weight. Values are expressed as means (*n* = 3) ± Standard Error Mean (SEM). Statistical differences were analyzed by two-way analysis of variances (ANOVA). Significant differences (^b^ *p* < 0.001, ^c^ *p* < 0.01 and ^d^ *p* > 0.05, followed by Bonferroni Posttests) compared with the control.

**Table 9:** GC-MS analysis of bioactive compounds produced by *Pseudomonas aeruginosa* A7.

RT	Major Compounds	MM	MF	Activity	Ref.
22.04	Oleic acid	282	C_18_H_32_O_2_	Unknown	
	4-fluoro-1-methyl-5-carboxylic acid	172	C_7_H_9_FN_2_O_2_	Unknown	
	2-tridecenoic acid	212	C_7_H_9_FN_2_O_2_	Unknown	
	6,10-dimethyl-4-undecanol	200	C_13_H_28_O	Unknown	
24.65	1-pentadecene	200	C_15_H_30_	Unknown	
	1-tridecene	182	C_13_H_26_	Unknown	
	10-heneicosene (C, T)	294	C_21_H_42_	Unknown	
	1-hexadecene	224	C_16_H_32_	antibacterial activity	[101]
28.06	Tridecanoic acid, 12-methyl-ester	242	C_15_H_30_O_2_	Unknown	
	Hexadecanoic acid, methyl ester	270	C_17_H_34_O_2_	antioxidant, antibacterial	[102]
	Tridecanoic acid, methyl ester	228	C_14_H_28_O_2_	Unknown	
	Hexacosanoic acid, methyl ester	410	C_27_H_54_O_2_	Unknown	
29.70	5-Eicosene	280	C_20_H_40_	antimicrobial, anticancer	[101]
	E-15-heptadecenal	252	C_17_H_32_O	antibacterial activity	
	Cycloeicosane	280	C_20_H_40_	antimicrobial, anticancer	
	1-heptadecene	238	C_17_H_34_	Unknown	
32.08	9-octadecenoic acid	296	C_19_H_36_O_2_	antibacterial activity	[103]
	12-octadecenoic acid	296	C_18_H_34_O_2_	Unknown	
	Trans-13-octadecenoic acid	238	C_18_H_34_O_2_	Unknown	
40.04	1,2-benzenedicarboxylic acid mono (2-ethylhexyl) ester	278	C_16_H_22_O_4_	antimicrobial, anticancer	[100]
	6-ethyloct-3-yl 2-ethylhexyl ester	418	C_26_H_42_O_4_	Unknown	
	1,2-benzenedicarboxylic acid diisooctyl ester, Bis (2-ethylhexyl) phthalate	390	C_24_H_38_O_4_	antifungal	[104]

Bacterial bioactive compounds produced by the bacterial strain were analyzed using Gas Chromatography–Mass Spectroscopy and the compounds were identified by the National Institute of Standards and Technology (NIST) library based on their retention time, molecular mass, and molecular formula. RT = Retention time, MM = Molecular mass, MF = Molecular formula. Ref. = References.

## Data Availability

All data related to this manuscript are incorporated in the manuscript only.

## Supplementary Materials

The following supporting information can be downloaded at: https://www.mdpi.com/article/10.3390/microorganisms10030568/s1, Figure S1: Bar diagram showing the production of Indole-3-acetic acid in the presence (5 mM) and absence of L-tryptophan by the bacterial strain A7 with 48 h time interval for 196 h, Figure S2A: *In vivo* establishment and control of Fusarium wilt disease of chickpea caused by *Fusarium oxysporum* f. sp. *ciceris* by the bacterial strain A7 at the first day (Day-1) of inoculation, Figure S2B: *In vivo* establishment and control of Fusarium wilt disease of chickpea caused by *Fusarium oxysporum* f. sp. *ciceris* by the bacterial strain A7 at 20th days (Day-20) of inoculation (a,b), Figure S2C: *In vivo* establishment and control of Fusarium wilt disease of chickpea caused by *Fusarium oxysporum* f. sp. *ciceris* by the bacterial strain A7 at 40th days (Day 40) of inoculation (a,b).
