# Supplementary material for: An Evaluation of Aluminum Tolerant Pseudomonas aeruginosa A7 for In Vivo Suppression of Fusarium Wilt of Chickpea Caused by Fusarium oxysporum f. sp. ciceris and Growth Promotion of Chickpea
Source: Microorganisms. 2022 Mar 5;10(3):568. doi: 10.3390/microorganisms10030568 (PMC8950562; doi:10.3390/microorganisms10030568)
Supplement: Supplementary file 1 [file microorganisms-10-00568-s001.zip › Supplementary figure S1.pdf]

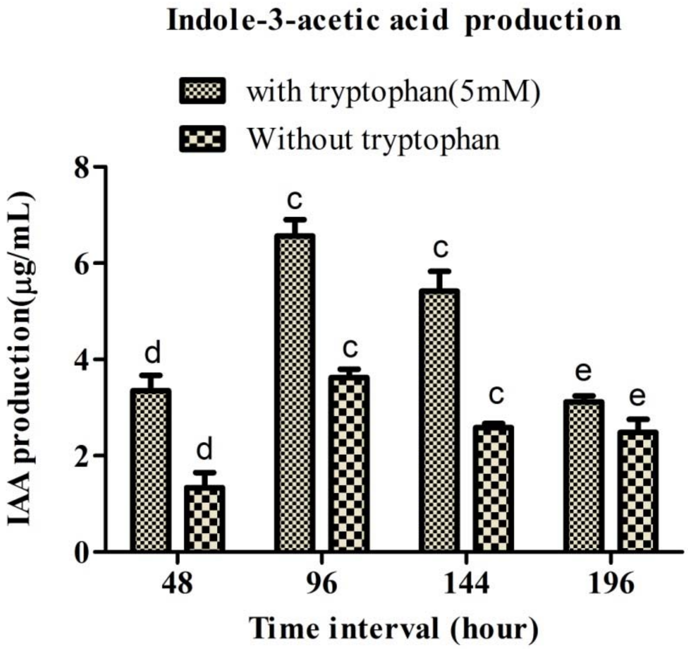

Figure S1. Bar diagram showing the affect of production of Indole -3- acetic acid in presence (5 mM) and absence of L-tryptophan on by the bacterial strain A7 with 48 h time interval for 196 hours. Values are expressed as means n = 3 ± Standard Error Mean (SEM). Statistical differences were analyzed by two-way analysis of variances (ANOVA). Significant differences (followed by Bonferroni Posttests) compared with the presence and absence of L-tryptophan are indicated by letters c, d and e.
