# Supplementary material for: An Evaluation of Aluminum Tolerant Pseudomonas aeruginosa A7 for In Vivo Suppression of Fusarium Wilt of Chickpea Caused by Fusarium oxysporum f. sp. ciceris and Growth Promotion of Chickpea
Source: Microorganisms. 2022 Mar 5;10(3):568. doi: 10.3390/microorganisms10030568 (PMC8950562; doi:10.3390/microorganisms10030568)
Supplement: Supplementary file 1 [file microorganisms-10-00568-s001.zip › Supplementary figure S2C.pdf]

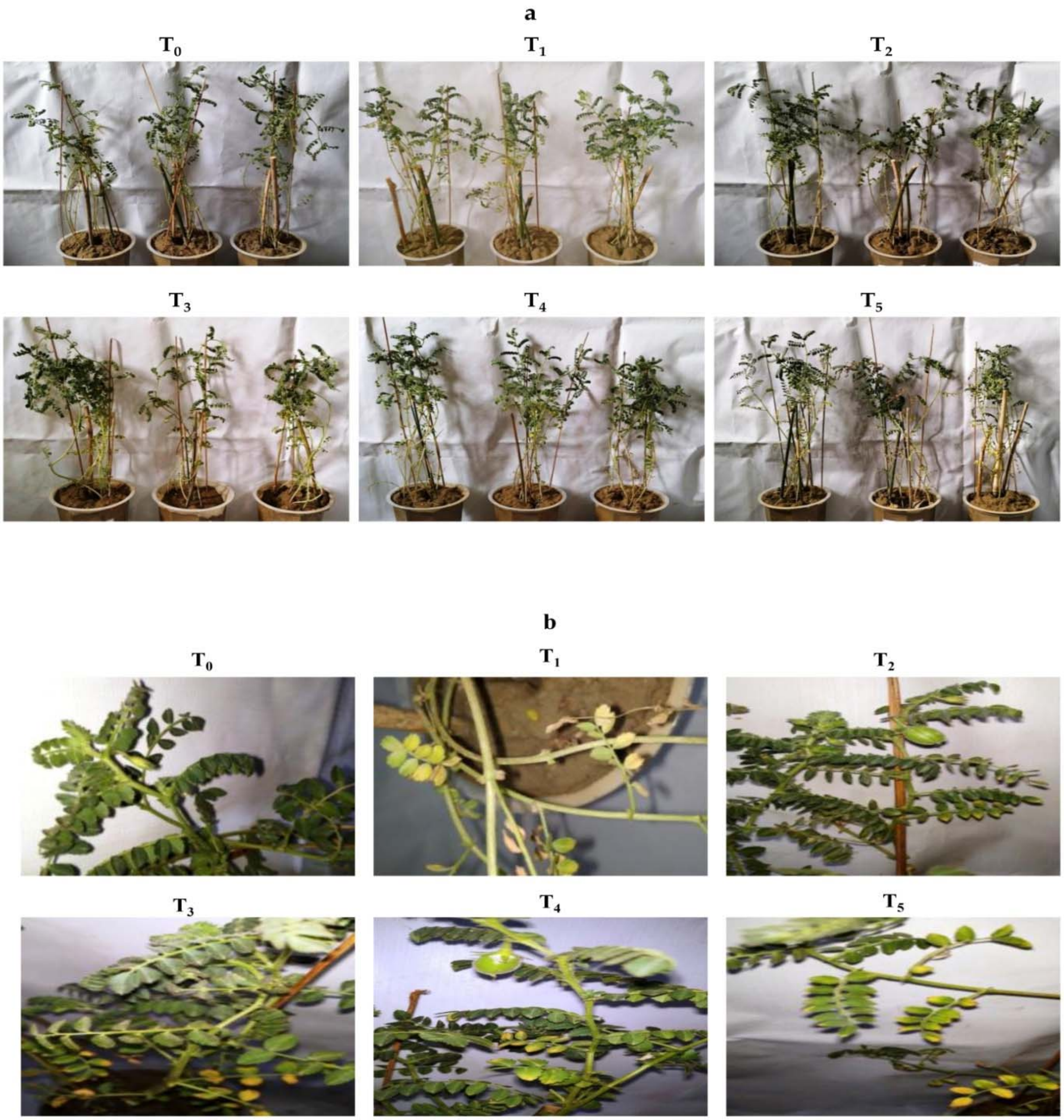

Figure S2C. *In vivo* establishment and control of Fusarium wilt disease of chickpea caused by *Fusarium oxysporum* sp. f. *ciceris* by the bacterial strain A7 at 40th days (Day 40) of inoculation (a-b) with the treatments as T_0 = Sterile distilled water as control, T_1 = Only bacterial cell suspension, T_2 = Only fungal spore suspension, T_3 = Bacterial cell suspension and fungal spore suspension, T_4 = Bacterial cell suspension two days before the fungal spore suspension and T_5 = Fungal spore suspension two days before the bacterial cell suspension at room temperature in three biological replicates.
